# Experimental Investigation on Hybrid Steel–Grout Vertical Joints with Steel Anchor Rings for Precast Concrete Shear Walls

**DOI:** 10.3390/ma19163424

**Published:** 2026-08-12

**Authors:** Zongchang Li, Bo Cui, Xiaolei Han, Zixiang Peng, Zinan Wu

**Affiliations:** 1 School of Civil Engineering & Transportation, South China University of Technology, Guangzhou 510641, China; 202411082222@mail.scut.edu.cn (Z.L.); 202421008624@mail.scut.edu.cn (B.C.); xlhan@scut.edu.cn (X.H.); 2 China Construction Fourth Engineering Bureau Corp., Ltd., Guangzhou 510665, China; 20237295@cscec.com

**Keywords:** precast concrete shear wall, vertical joint, steel-anchor-ring grouted connection, shear resistance

## Abstract

Vertical joints are commonly required in precast concrete shear walls when large wall panels are divided for fabrication, transportation, and erection. To reduce on-site work and improve assembly efficiency, this study proposes and investigates a hybrid steel–grout vertical joint, namely a steel-anchor-ring grouted connection, which uses embedded threaded sleeves, steel anchor rings, an inserted vertical bar, and high-strength grout to transfer shear between adjacent wall panels. Monotonic direct shear tests, cyclic shear tests under normal tension and compression, and cyclic loading tests on precast shear wall specimens were conducted to investigate its mechanical behavior. The load–slip behavior, observed interface debonding, and final shank fracture in the direct shear tests suggested that grout–panel interface bond contributed substantially to the pre-peak response, whereas anchor-ring action became increasingly important after bond degradation. Comparison of the cyclic shear specimens suggested that normal tension promoted interface opening and bond degradation, leading to slip-dominated behavior, whereas normal compression enhanced interface contact and post-peak stability. For the specimens tested, the measured-to-calculated resistance ratios based on the code-based expression were 1.97–2.70 under direct shear and compression–shear conditions, but decreased to 1.20–1.31 under tension–shear loading. Wall-level demand analysis showed that the shear transferred across the vertical joint was significantly affected by both joint location and wall shear span ratio; larger shear span ratios increased the joint shear demand, whereas offset joint layouts reduced the demand. The wall specimens, designed relative to the code-based resistance, exhibited limited joint-related cracking and stable hysteretic behavior, and failed by wall-base flexural damage, indicating the shear-transfer effectiveness of the proposed joint under the tested configurations.

## 1. Introduction

### 1.1. Research Background

Precast concrete structures have been increasingly adopted because factory production improves quality control, accelerates construction, reduces on-site labor, and limits wet work [1,2,3,4]. Existing studies on precast beams, columns, and beam–column joints have developed various connection techniques to restore member-end continuity [5,6,7,8,9]. However, precast shear walls present a different connection problem because they are large two-dimensional lateral-force-resisting components rather than one-dimensional members connected mainly at their ends. They are often divided into panels because of fabrication, transportation, lifting, and erection constraints. After panelization, joints become part of the wall configuration and influence inter-panel shear transfer, deformation compatibility, and integrated lateral resistance. Dense reinforcement in thin wall sections further increases alignment, grouting, and construction-tolerance challenges [10,11,12,13,14,15].

Precast shear wall systems can generally be developed through two routes: composite or superposed systems and panelized systems assembled by wall-to-wall joints [16]. Composite or superposed walls, including double-skin walls, double-face superposed walls, and walls with precast shells and post-cast cores, use precast components as a permanent formwork and achieve structural integrity through cast-in-place concrete in the middle region or local joint zones [17,18]. These systems can improve construction quality and reduce formwork, but still require on-site concrete casting, reinforcement arrangement, and curing. To further reduce wet work and improve assembly efficiency, panelized precast shear walls have attracted increasing interest. In these systems, complete wall panels are connected through localized horizontal and vertical joints. Horizontal joints connect upper and lower wall panels or wall panels to foundations, and are mainly designed to transfer vertical reinforcement forces, flexural compression and tension, and interface shear. Existing horizontal joint methods may be broadly classified according to the connection principle as wet, dry, and hybrid connections. Wet connections, such as grouted sleeves, grouted ducts, reserved-hole grouted lap splices, and post-cast joints, aim to emulate cast-in-place behavior through reinforcement continuity and grouted or cast-in-place regions [19,20,21]. However, their construction quality depends on bar alignment, sleeve or duct arrangement, anchorage length, and grout compactness, which can be challenging in thin wall sections with dense reinforcement [22,23]. Dry connections using bolted plates or steel connectors reduce wet work and provide a mechanical load path, but require control of connector slip, local bearing, and installation tolerance [24]. Dry mechanical connections have also been explored in demountable modular RC wall systems, where replaceable precast panels are assembled using bolted joints to improve damage control and post-earthquake reparability [25]. Hybrid or steel-assisted details combine mechanical connectors with local grouting or post-cast materials to balance force transfer, construction tolerance, and assembly efficiency [26,27,28]. Therefore, horizontal joint studies provide a useful reference for connection principles, while vertical joints between adjacent wall panels require separate consideration because their role is to ensure inter-panel shear transfer along the wall length.

Vertical joints deserve separate attention because they maintain composite action between wall panels placed side by side. When a large wall is divided along its length, the joint must transfer in-plane shear flow between adjacent panels so that the assembled wall can act as an integrated lateral-force-resisting component. Therefore, vertical joints are not merely construction details; their strength, stiffness, and deformability affect shear distribution, deformation compatibility, and wall-level response. Studies on large-panel precast wall buildings have shown that vertical joint behavior directly influences inter-panel interaction and should be considered in analysis and design [29,30,31,32].

Early studies mainly focused on grout-filled, reinforcement-based vertical joints between precast wall panels. These studies investigated joint surface geometry, such as plane, castellated, or keyed interfaces, and transverse connection details, such as U-bars, wire loops, and looped reinforcement crossing the grouted region [29,30,32]. They showed that shear transfer is governed by the combined action of grout–panel interaction and transverse connectors, rather than reinforcement continuity alone [33,34,35,36]. The grout–panel interface and joint geometry contribute bond, contact resistance, mechanical interlock, and diagonal compression paths, whereas transverse connectors provide dowel action, crack bridging, confinement, and residual resistance after interface cracking. Normal stress may further alter this mechanism: compression can enhance contact and diagonal compression transfer, while tension may promote interface opening, bond degradation, and sliding failure [35,36,37]. Thus, grouted vertical joints should be assessed by distinguishing grout–panel interaction from connector resistance, with consideration of possible normal-stress effects.

Although wet grouted vertical joints can provide reliable shear transfer and emulate cast-in-place behavior, their construction details remain demanding. U-bars, looped bars, and lap-spliced details usually require side-face projecting reinforcement, sufficient overlap length, transverse confinement, and a post-cast or grouted joint region. These requirements may enlarge the joint width, increase the amount of site-placed concrete, and introduce on-site bar binding, formwork, casting, and curing operations. They may also create rebar congestion and reduce the tolerance for component adjustment during erection, particularly when protruding or pre-embedded bars restrict the relative movement of adjacent panels [37,38,39]. To reduce site casting and protruding reinforcement, dry mechanical vertical joints using welded plates, bolted plates, embedded steel connectors, or box connectors have also been developed [40]. These details provide a direct mechanical load path and can reduce wet work. However, their shear transfer is mainly concentrated in discrete steel connectors, and the wall-level composite action therefore depends on the stiffness, strength, and deformation capacity of these local connectors [10,13,24]. Connector slip, local bearing deformation, bolt loosening, or weld-related defects may weaken the stability of shear transfer under cyclic loading. This limitation has motivated hybrid solutions in which steel components are combined with grout filling, bearing, or clamping effects.

A limited number of hybrid connections for vertical joints have been proposed. Menegon et al. [41] developed grouted panel pocket and post-tensioned corbel connections as hybrid alternatives to welded stitch plates and wet joints in precast building cores. These connections showed higher strength and stiffness than the baseline welded stitch plate connection. However, their hybrid action is achieved through localized prefabricated connection modules. The wall-level composite action is therefore controlled by the stiffness, strength, and deformation capacity of these local modules. From a construction perspective, the paired pockets, corbel regions, embedded receiving components, and inserted connectors must be accurately fabricated and aligned in adjacent panels so that the connector from one panel can properly engage with the corresponding region in the other panel after erection. This increases fabrication difficulty and makes assembly sensitive to inter-panel positional mismatch. Therefore, although existing hybrid connections can reduce conventional wet-joint or site-welding operations, their local-module strategy still involves concentrated shear transfer and strict panel-to-panel matching during erection. This leaves a need for a hybrid vertical joint in which the steel–grout mechanism is distributed along the joint height while retaining sufficient installation adjustability.

### 1.2. Proposed Hybrid Steel–Grout Joint and Research Scope

To address this need, this study proposes a steel-anchor-ring grouted vertical joint for precast concrete shear walls. In the proposed joint, steel anchor rings are arranged at multiple levels along the vertical joint and linked by an inserted vertical bar within a grouted joint region, forming a distributed hybrid steel–grout shear-transfer mechanism along the joint height. From the fabrication perspective, the joint-side faces of the precast panels are intentionally designed as smooth surfaces, with only embedded threaded sleeves and local operation openings provided inside the panels. Therefore, side-face projecting reinforcement and large matching recesses are avoided, facilitating panel molding, demolding, storage, transportation, and lifting. From the assembly perspective, the steel anchor rings are installed after panel erection and can be adjusted before the inserted vertical bar is placed. As a result, the proposed joint does not require two panels to have fully matched local receiving modules before erection; the critical alignment can be adjusted and verified during installation. These construction and mechanical features, together with those of representative wet, dry mechanical, and existing hybrid vertical joints, are summarized in Table 1.

**Figure 1:**
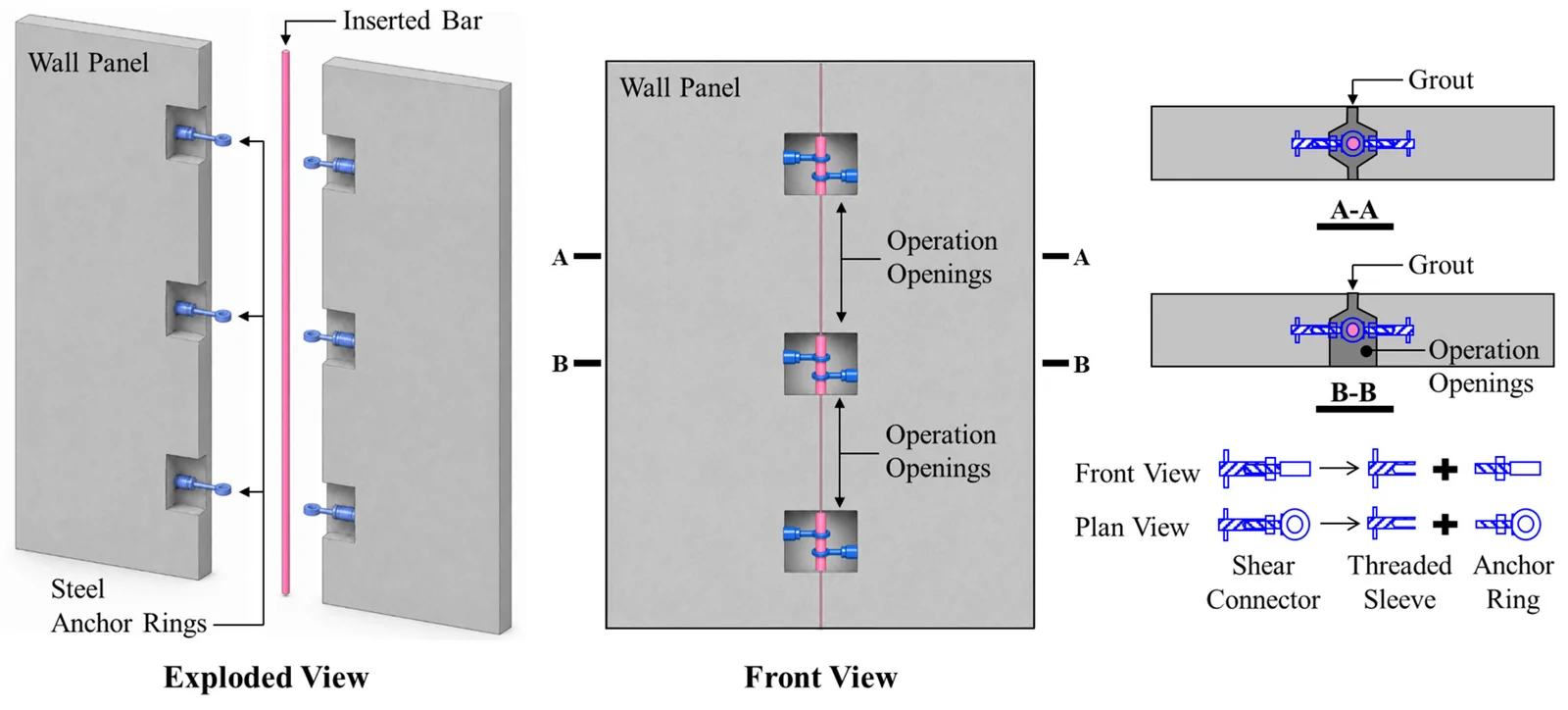
Steel-anchor-ring grouted vertical joint.

As shown in Figure 1, threaded sleeves are pre-embedded in the side faces of the precast wall panels, and local operation openings are reserved for installation and grouting. During panel fabrication, the threaded sleeves were fixed at their designated positions and checked before concrete casting to minimize positional deviations during concrete placement and vibration. Before panel erection, the joint-side surfaces that would form the grouted cavity, together with the surfaces within the operation openings, were cleaned to remove dust, loose particles, and other contaminants. During assembly, the adjacent panels were erected, temporarily supported, and adjusted to the target joint width and vertical alignment.

The steel anchor rings were then manually screwed into the embedded sleeves through the operation openings. The thread engagement length was not less than three times the anchor-ring shank diameter. The external projection and rotational position of each anchor ring were adjusted until the ring openings were vertically oriented and their centers were aligned along the joint height. The locking nuts were then tightened manually to secure the adjusted positions, although the tightening torque was not measured. Finally, the inserted vertical bar was passed through all ring openings without forcing, which served as a practical check of the cumulative alignment of the embedded sleeves, wall panels, and steel anchor rings.

Before grouting, the accessible surfaces inside the operation openings and joint cavity were visually checked again, and loose particles generated during installation were removed where necessary. The operation openings used in the joint-level specimens provided access for anchor-ring installation, vertical-bar insertion, grout placement, and visual inspection. The joint cavity and operation openings were subsequently filled with high-strength cementitious grout, with particular attention given to avoiding voids and incomplete filling. The grout-filled joint region, steel anchor rings, and inserted vertical bar thereby formed a continuous shear-transfer path between the adjacent wall panels.

No major difficulty was encountered during laboratory assembly when the embedded sleeves and wall panels were positioned within the intended geometry. The principal construction-control points were the positional accuracy of the embedded sleeves and the available working clearance within the operation openings. Sleeve displacement during concrete casting or excessive panel misalignment could prevent alignment of the anchor-ring openings and obstruct insertion of the vertical bar. Insufficient clearance within the operation openings could also restrict manual rotation and tightening of the anchor rings. These conditions were therefore checked and corrected before grouting.

The objective of this study is to clarify the shear behavior and applicability of the proposed joint. Section 2 and Section 3 present the joint-level tests, including monotonic direct shear tests and cyclic shear tests under different normal stress states. These tests were designed to identify the damage patterns, resistance mechanism, and the effect of normal stress on joint shear transfer. Based on the joint-level results, the measured peak resistances were compared with selected code-based and interface-shear expressions. Section 4 presents elastic wall-demand estimates and cyclic tests on precast shear wall specimens to demonstrate the performance of the vertical joints under the tested demand levels.

## 2. Joint-Level Experimental Program

### 2.1. Overview of Joint-Level Tests

A joint-level experimental program was conducted to investigate the shear behavior of steel-anchor-ring grouted vertical joints under monotonic and cyclic loading conditions. The joint-level specimens were designed to represent the local shear-transfer region of a vertical joint between adjacent precast wall panels. Two types of tests were included: direct shear tests under monotonic loading and cyclic shear tests under different normal stress conditions.

The direct shear tests were mainly used to identify the monotonic shear load–joint slip response, cracking process, peak resistance, and post-peak behavior of the joint. The cyclic shear tests were conducted to examine the influence of normal tension and compression on the cyclic shear response, with emphasis on skeleton-curve characteristics and hysteretic-loop shapes. In total, five joint-level specimens were tested, including three direct shear specimens and two cyclic shear specimens.

### 2.2. Specimen Design

**Figure 2:**
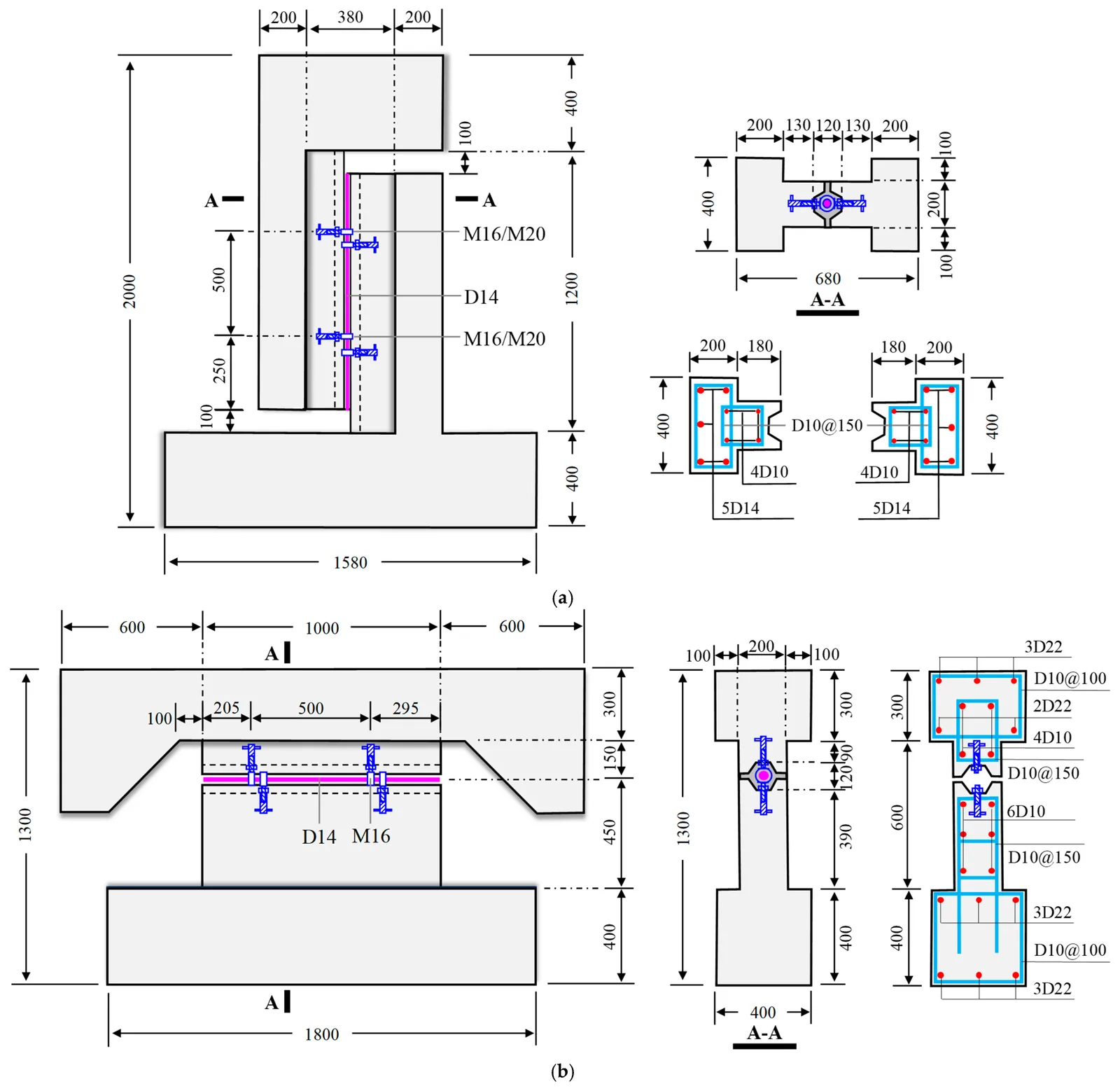
Geometric details and connection arrangement of the joint-level specimens: (**a**) direct shear specimen; (**b**) cyclic shear specimen.

Each joint-level specimen consisted of two precast wall-panel segments connected through steel anchor rings, an inserted vertical bar, and high-strength grout. Figure 2 shows the geometric details and connection arrangement of the joint-level specimens, including the direct shear specimen and the cyclic shear specimen.

For the direct shear specimens, the joint region was arranged vertically, as shown in Figure 2a, so that monotonic shear loading could be applied parallel to the joint. Three direct shear specimens were designed, namely DS16-C40, DS20-C40, and DS16-C30, to examine the local shear behavior of the grout-panel interfaces and the anchor-ring connection system. DS16-C40 was used as the reference specimen, adopting M16 steel anchor rings and C40 concrete panels. Compared with DS16-C40, DS20-C40 used M20 steel anchor rings to investigate the effect of anchor-ring size, whereas DS16-C30 used C30 concrete panels to investigate the effect of panel concrete strength.

For the cyclic shear specimens, the configuration was modified to apply a constant normal force perpendicular to the joint together with reversed shear loading parallel to the joint, as shown in Figure 2b. Joint-normal forces arise from deformation incompatibility between adjacent components. These effects are generally more pronounced in wall-to-column connections because the wall and column exhibit different lateral deformation characteristics [37]. By contrast, in wall-to-wall connections, the adjacent wall panels generally deform compatibly, and the vertical joint primarily transfers vertical shear flow between the panels [29,30]. Consequently, the resultant joint-normal force is typically limited. Accordingly, relatively low normal-force levels were adopted. Specimen TS16 was subjected to a constant normal tensile force of −20 kN, whereas specimen CS16 was subjected to a constant normal compressive force of 40 kN. Both specimens adopted M16 steel anchor rings and C40 concrete panels.

For all joint-level specimens, the wall-panel thickness was 200 mm, the joint length was 1000 mm, the grouted-hole diameter was 120 mm, and the inserted vertical bar had a diameter of 14 mm. The steel anchor rings were arranged at a vertical spacing of 500 mm. The same type of high-strength cementitious grout was used for all grouted holes. The specimen details are summarized in Table 2.

### 2.3. Material Properties for Joint-Level Tests

Material tests were carried out to determine the mechanical properties of the concrete, grout, steel anchor rings, and inserted bars used in the joint-level specimens. For each concrete grade, three 150 mm × 150 mm × 300 mm prismatic specimens were cast from the same batch as the corresponding joint specimens, cured under standard conditions, and tested at 28 days in accordance with GB/T 50081-2019 [42]. The mean compressive strengths *f*_c_ of the C30 and C40 concrete were 22.1 MPa and 32.8 MPa, respectively, with corresponding coefficients of variation of 12% and 11%. The splitting tensile strengths *f*_t_, determined from three companion 150 mm cubic specimens for each concrete grade, were 2.4 MPa and 3.3 MPa, respectively.

Accordingly, the applied normal compressive force in Specimen CS16 corresponded to a normal compression stress ratio of 0.006, calculated with respect to *f*_c_, whereas the applied normal tensile force in Specimen TS16 corresponded to a normal tension stress ratio of 0.03, calculated with respect to *f*_t_. Both ratios were relatively low, consistent with the limited joint-normal force expected in wall-to-wall connections.

The high-strength cementitious grout used to fill the grouted holes and operation openings was characterized using three 40 mm × 40 mm × 160 mm prismatic specimens cast from the same batch, cured under standard conditions, and tested at 28 days in accordance with GB/T 17671-2021 [43]. The prisms were first subjected to flexural testing, after which the six resulting half-prisms were used for compressive testing. The mean compressive and flexural strengths of the grout were 66.1 MPa and 7.1 MPa, respectively.

The steel anchor rings were fabricated from 45# carbon steel, while the inserted vertical bars were D14 HRB400 deformed bars. For each steel type, three representative tensile specimens were tested at room temperature in accordance with GB/T 228.1-2021 [44]. An extensometer was used to record the longitudinal strain, and the yield strain, yield strength, and ultimate tensile strength were determined from the measured stress–strain responses. The coefficients of variation in the yield and ultimate tensile strengths were 0.77% and 0.71%, respectively, for the steel anchor rings, and 0.60% and 0.51%, respectively, for the inserted bars. The mean measured properties are summarized in Table 3.

### 2.4. Test Setup

**Figure 3:**
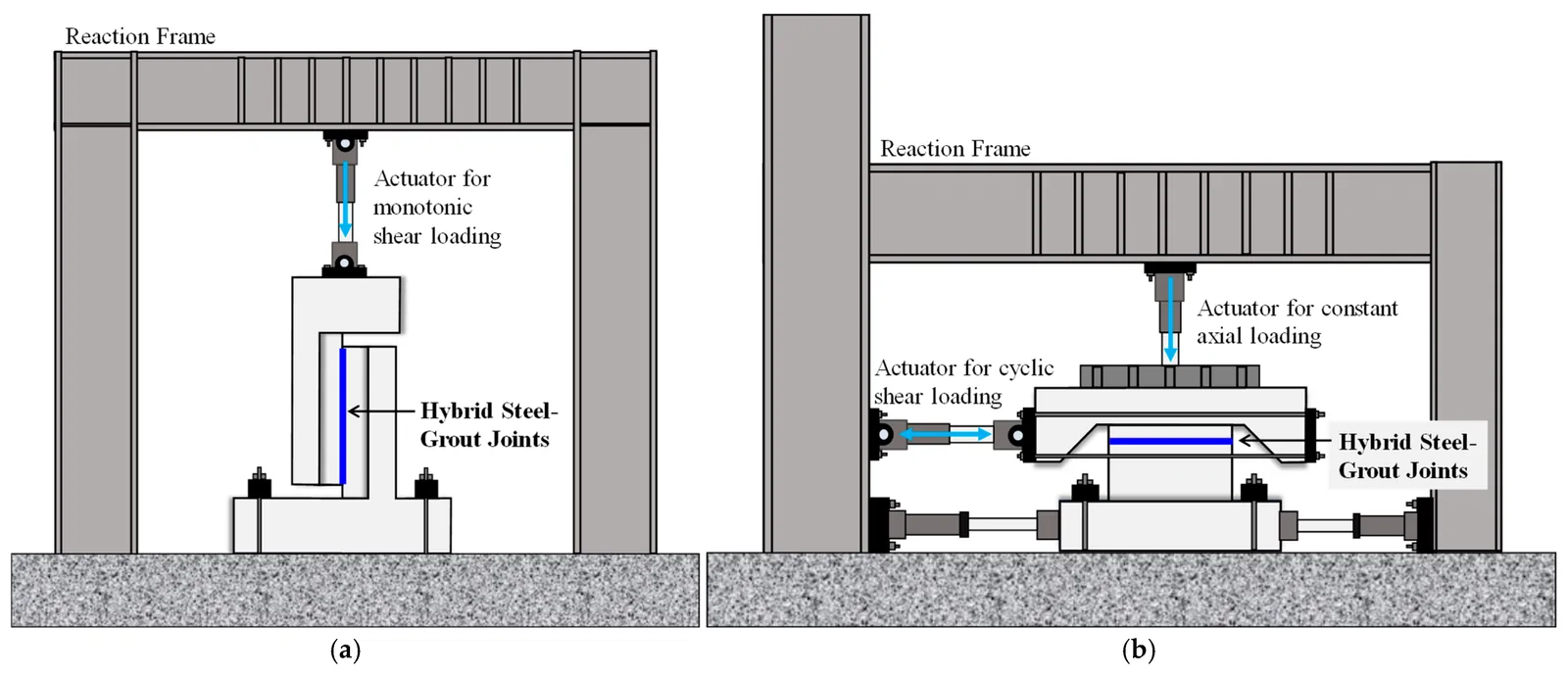
Test setups for the joint-level specimens: (**a**) direct shear test; (**b**) cyclic shear test.

The test setups for the joint-level specimens are shown in Figure 3, including the direct shear setup, the tension–shear setup, and the compression–shear setup.

For the direct shear tests, as shown in Figure 3a, the specimen was positioned vertically, and monotonic shear loading was applied parallel to the joint through the loading beam. The foundation beam was fixed to the laboratory floor to provide a stable support condition. This setup was used to evaluate the monotonic shear response of the joint without externally applied normal force.

For the cyclic shear tests, as shown in Figure 3b, a constant normal force was applied perpendicular to the joint, while reversed cyclic shear loading was applied parallel to the joint. Specimen TS16 was subjected to a tensile normal force to represent the tension–shear condition, whereas specimen CS16 was subjected to a compressive normal force to represent the compression–shear condition. This arrangement enabled the joint response to be examined under combined normal-force and cyclic shear actions.

The instrumentation was arranged to monitor both the global response of the specimen and the local deformation of the joint. The applied shear load and normal force were recorded throughout the tests. Displacement transducers were installed across the joint to measure the relative shear displacement between the two precast panel segments. This measured quantity is hereafter defined as the joint slip and was used as the horizontal coordinate of the joint-level response curves.

### 2.5. Loading Protocol

For the direct shear tests, monotonic loading was applied in two stages. At the initial stage, load control was adopted to capture the elastic response and the onset of visible cracking. After the response became nonlinear, displacement control was used to trace the post-cracking and post-peak behavior until fracture of the steel-anchor-ring shanks occurred.

For the cyclic shear tests, the target normal force was first applied perpendicular to the joint and was maintained approximately constant during the subsequent shear-loading process. Reversed cyclic shear loading was then applied parallel to the joint using a two-stage protocol. In the first stage, force control was adopted. The shear load was increased in increments of 20 kN, and one cycle was imposed at each load level until the load reached 100 kN. In the second stage, the loading mode was switched to displacement control. The target displacement was increased in increments of 2 mm, and two cycles were applied at each displacement level. The test was continued until fracture of the steel-anchor-ring connection occurred.

## 3. Joint-Level Experimental Results

This section presents the joint-level experimental results of the steel-anchor-ring grouted vertical joints. The direct shear response is first analyzed in terms of damage development, shear load–joint slip behavior, and force-transfer mechanism. The cyclic shear response is then examined to clarify the influence of normal tension and compression on cracking pattern, hysteretic behavior, peak resistance, and post-peak degradation. Based on these results, the shear resistance of the tested joints is further evaluated using a code-based expression, with emphasis on the variation in the measured-to-calculated resistance ratio under different normal-force conditions.

### 3.1. Direct Shear Response

#### 3.1.1. Damage Patterns Under Direct Shear Loading

**Figure 4:**
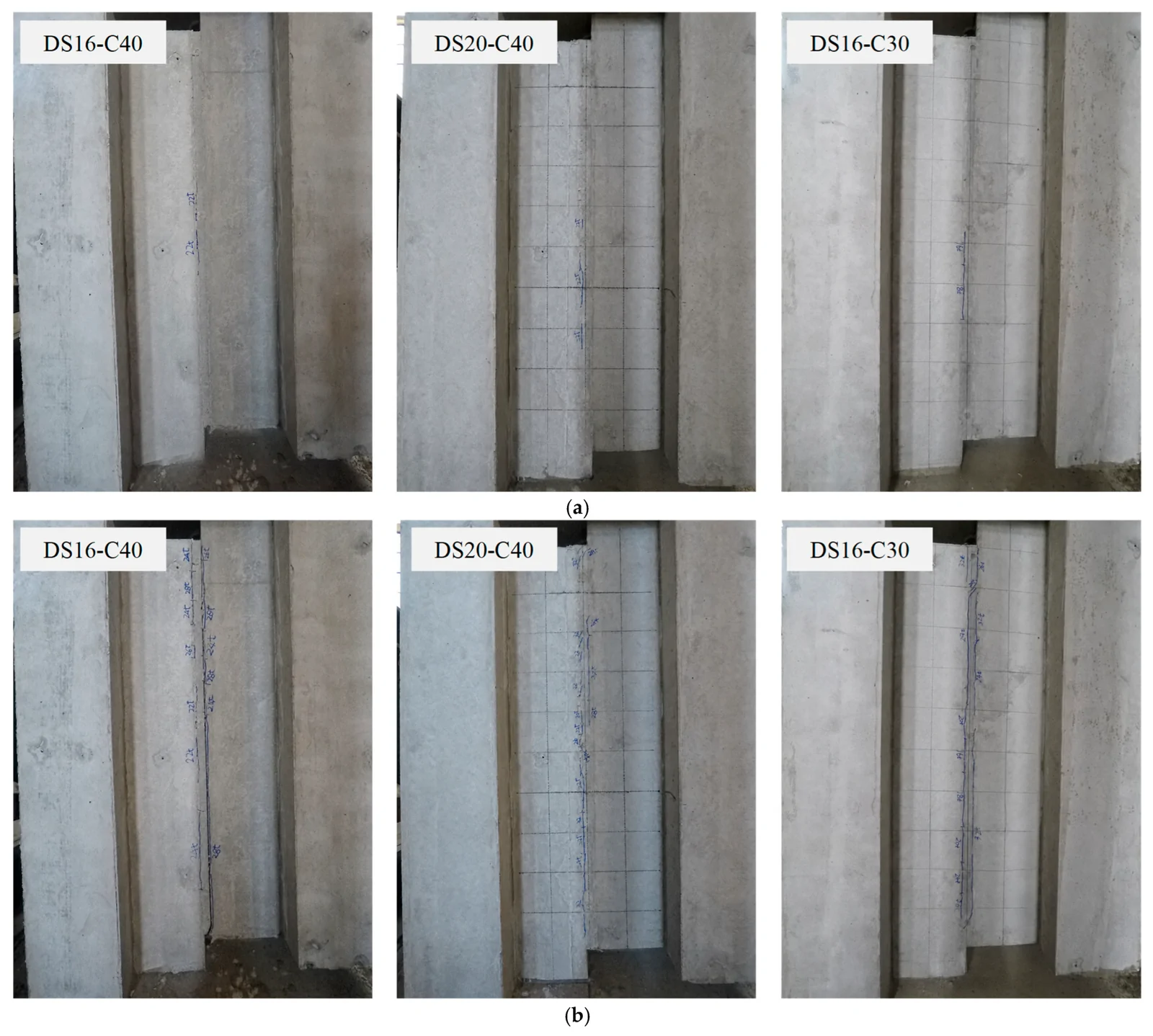

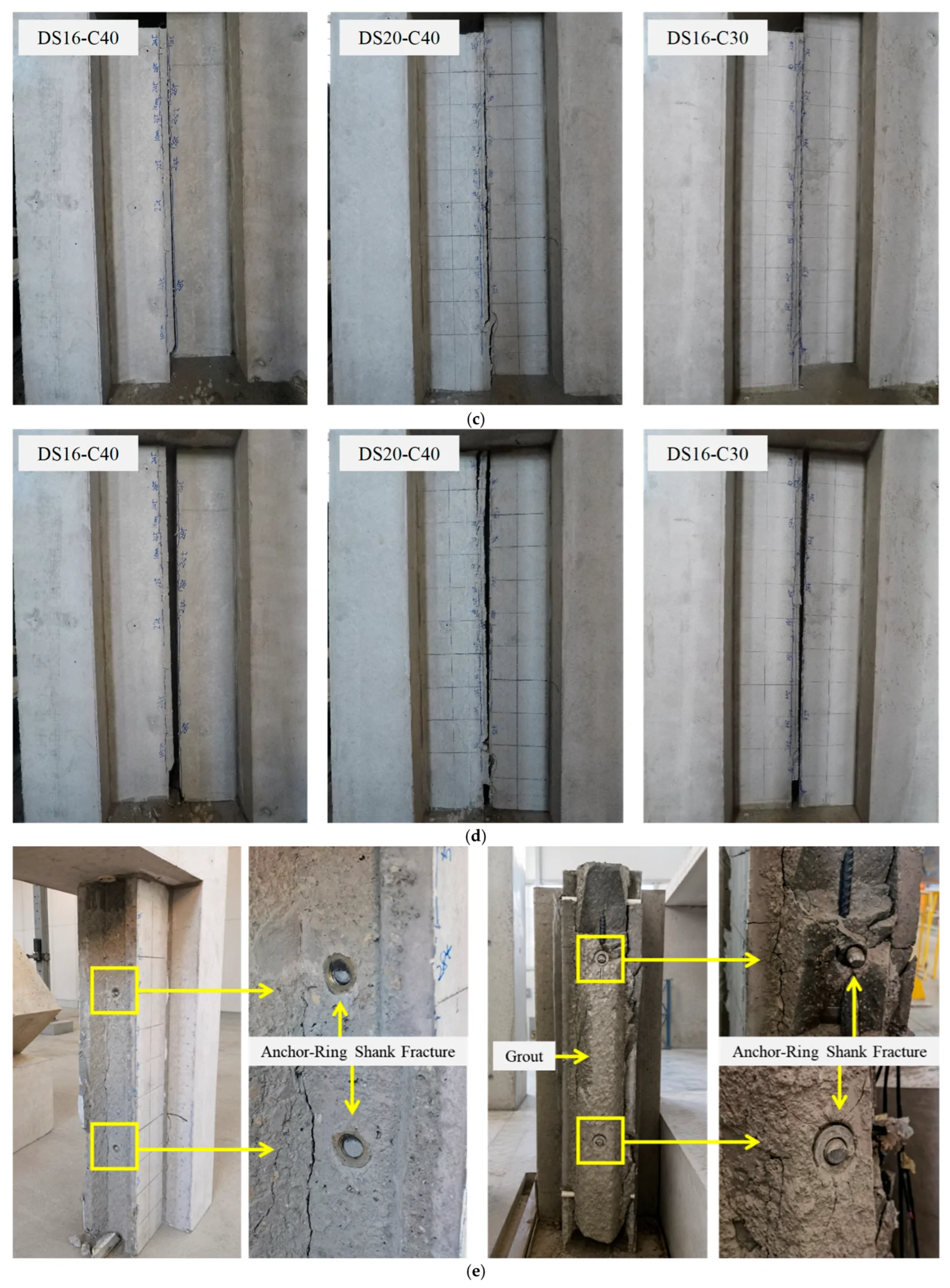
Damage patterns of direct shear specimens: (**a**) initial cracking; (**b**) cracking at peak load; (**c**) debonding; (**d**) final failure; (**e**) fracture surface and details.

**Figure 5:**
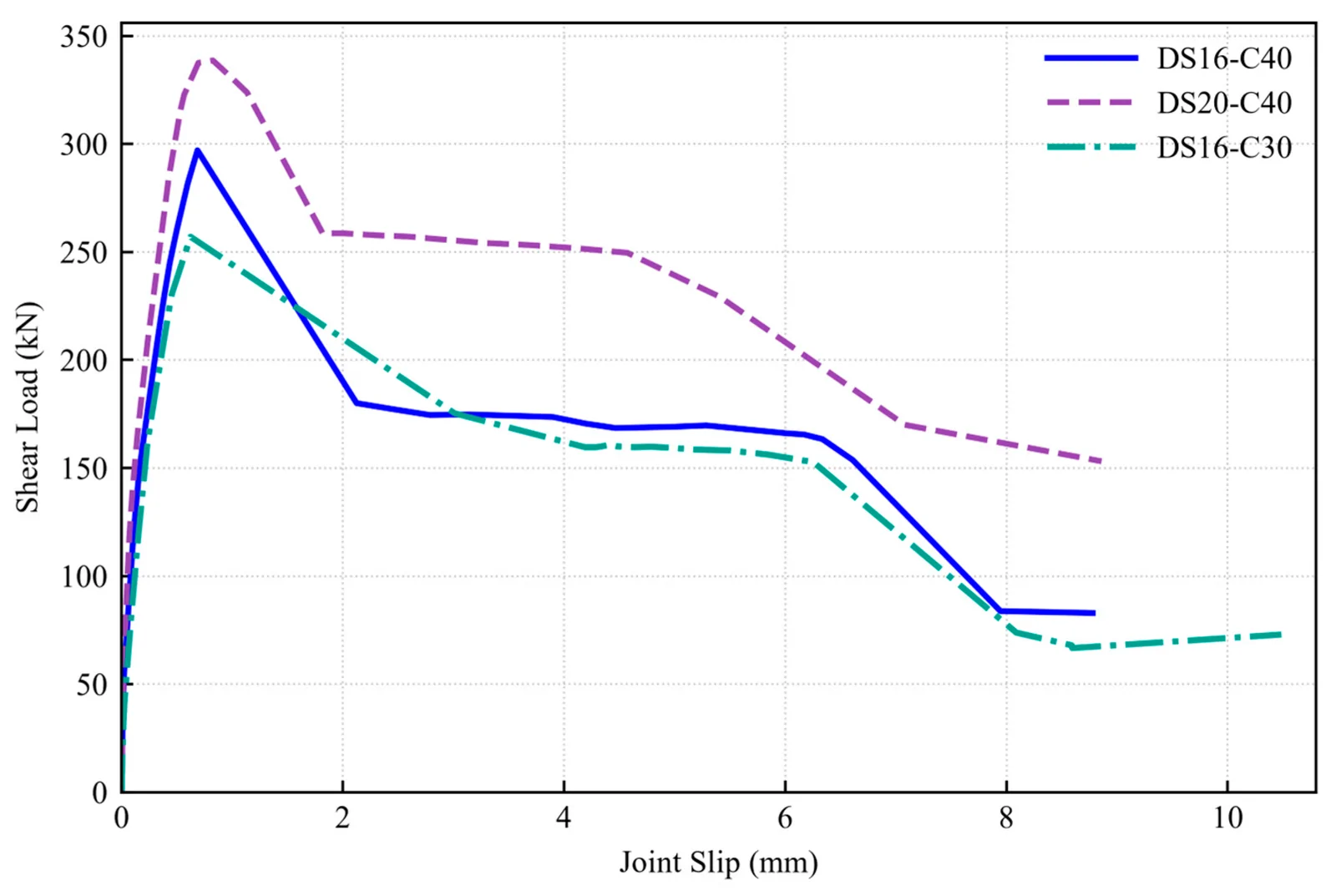
Shear load–joint slip curves of direct shear specimens.

Figure 4 and Figure 5 show the damage patterns and shear load–joint slip curves of the direct shear specimens, respectively. The three specimens exhibited a similar damage sequence, including interface cracking, crack propagation along the grout–panel interfaces, increasing joint slip, post-peak resistance degradation, residual resistance, and final fracture of the steel-anchor-ring shanks.

At the early loading stage, no visible damage was observed along the vertical joint, and the shear response increased approximately linearly. The approximately linear response before visible cracking was interpreted as being associated mainly with intact grout–panel interface bond, while the steel anchor rings restrained relative movement between the two precast panel segments. As the shear load increased, visible cracking first appeared near the middle of one grout–panel interface, as shown in Figure 4a. The initial crack subsequently extended over most of the interface, while cracking also developed along the opposite interface.

Near the peak load, damage became concentrated along the grout–panel interfaces. As shown in Figure 4b, a nearly continuous crack formed along the interface, accompanied by audible cracking and visible relative slip between the two precast panel segments. The shear load–joint slip curve simultaneously deviated from its initial linear response, which was consistent with progressive degradation of the grout–panel interface bond. The increasing joint slip was interpreted as progressively mobilizing the mechanical action of the steel anchor rings, allowing the joint to maintain its integrity after interface cracking.

After the peak load, interface debonding became more pronounced and the joint slip increased rapidly. As shown in Figure 4c, localized relative displacement developed along the joint, accompanied by minor spalling or crushing near the grout–panel interfaces and anchor-ring regions. The combination of pronounced interface debonding, sustained post-peak resistance, and eventual shank fracture was interpreted as indicating an increasing contribution from the mechanical action of the steel anchor rings. However, because local connector strains and force redistribution were not measured, the relative contributions of interface interaction and anchor-ring action cannot be quantified directly.

The specimens therefore entered a residual-resistance stage instead of losing their shear resistance immediately after the peak. With further increase in joint slip, local grout splitting developed around the anchor-ring regions, followed by fracture of the anchor-ring shanks, as shown in Figure 4d,e. The shank fracture caused a sharp reduction in shear resistance and governed the final failure of the joint.

#### 3.1.2. Shear Load–Joint Slip Responses

As shown in Figure 5, the shear load–joint slip curves were consistent with an inferred transition from a pre-peak response strongly influenced by grout–panel interface bond to a post-peak response with increasing reliance on anchor-ring action as interface degradation progressed.

The reference specimen DS16-C40 reached a peak shear resistance of 297 kN at a joint slip of 0.69 mm. Specimen DS20-C40 reached a higher peak resistance of 339 kN at a joint slip of 0.83 mm, representing an increase of 14.1%. DS20-C40 also maintained a higher residual-resistance level after the post-peak drop. For the tested comparison, increasing the anchor-ring size from M16 to M20 was therefore associated with increases in both peak and residual resistance.

Specimen DS16-C30 reached a peak resistance of 257 kN at a joint slip of 0.62 mm, which was 13.5% lower than that of DS16-C40. Because the two specimens had the same M16 anchor-ring arrangement, the lower peak resistance of DS16-C30 was consistent with a reduced interface-bond contribution associated with the lower panel concrete strength. Their relatively close residual-resistance branches suggest that the common M16 anchor-ring arrangement played an important role in the post-peak response after substantial interface degradation.

Overall, the load–slip responses, observed interface debonding, and final shank fracture suggest that the grout–panel interface bond contributed substantially to the pre-peak response, whereas anchor-ring mechanical action became increasingly important as interface degradation progressed. Lower panel concrete strength was associated mainly with lower peak resistance, whereas a larger anchor-ring size was associated with higher peak and residual resistance.

### 3.2. Cyclic Shear Response

#### 3.2.1. Damage Patterns Under Cyclic Shear Loading

**Figure 6:**
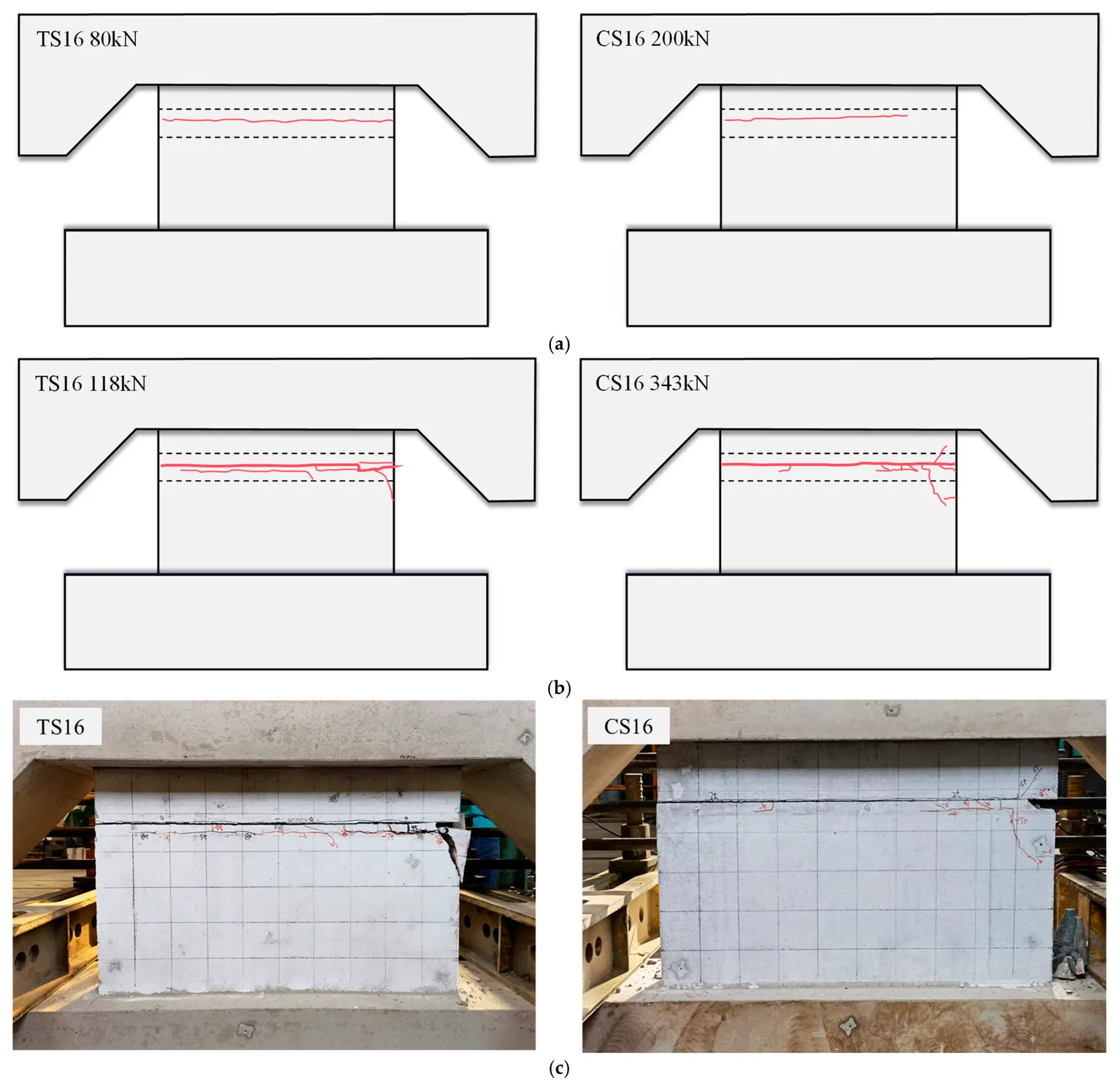
Damage patterns of cyclic shear specimens: (**a**) initial cracking; (**b**) cracking at peak load; (**c**) final failure.

Different from the direct shear specimens, the cyclic shear specimens were subjected to reversed shear loading while a constant normal force was maintained perpendicular to the joint. Figure 6 shows the damage patterns of the cyclic shear specimens.

The TS16 specimen was characterized by early two-sided interface cracking and slip-dominated damage. As shown in Figure 6a, visible cracking appeared at a relatively low shear load of 80 kN. When the peak resistance was reached, through cracks had formed along both the upper and lower grout-panel interfaces, indicating that the interface bond on both sides of the grout region had been substantially degraded. During displacement-controlled loading, these interface cracks continued to widen, and horizontal joint slip became increasingly pronounced. Partial fracture of the steel-anchor-ring shanks occurred at joint slips of around ±4 mm, accompanied by local grout crushing around the anchor-ring region, leading to a significant reduction in shear resistance. These observations indicate that normal tension weakened the interface bond at an early stage and accelerated the transition toward anchor-ring-dominated shear transfer.

By contrast, the CS16 specimen exhibited a distinct damage pattern, characterized by delayed and mainly one-sided interface cracking. As shown in Figure 6a, visible cracking appeared at a much higher shear load of around 200 kN. More importantly, when the peak resistance was reached, obvious cracking was mainly concentrated along one grout-panel interface, as shown in Figure 6b, whereas the opposite interface did not develop a through crack similar to that observed in TS16. This indicates that normal compression helped maintain interface contact and prevented simultaneous opening of the two grout-panel interfaces. During displacement-controlled loading, local corner spalling developed at joint slip of around ±4 mm, and fracture of the steel-anchor-ring shanks occurred at around ±8 mm. After anchor-ring fracture, the specimens still maintained a relatively stable residual resistance at larger joint shear-slip amplitudes, mainly due to friction along the compressed interface. Therefore, compared with the tension–shear specimens, the compression–shear specimens showed delayed cracking, less slip-dominated damage, and more stable post-peak resistance.

#### 3.2.2. Hysteretic Responses

**Figure 7:**
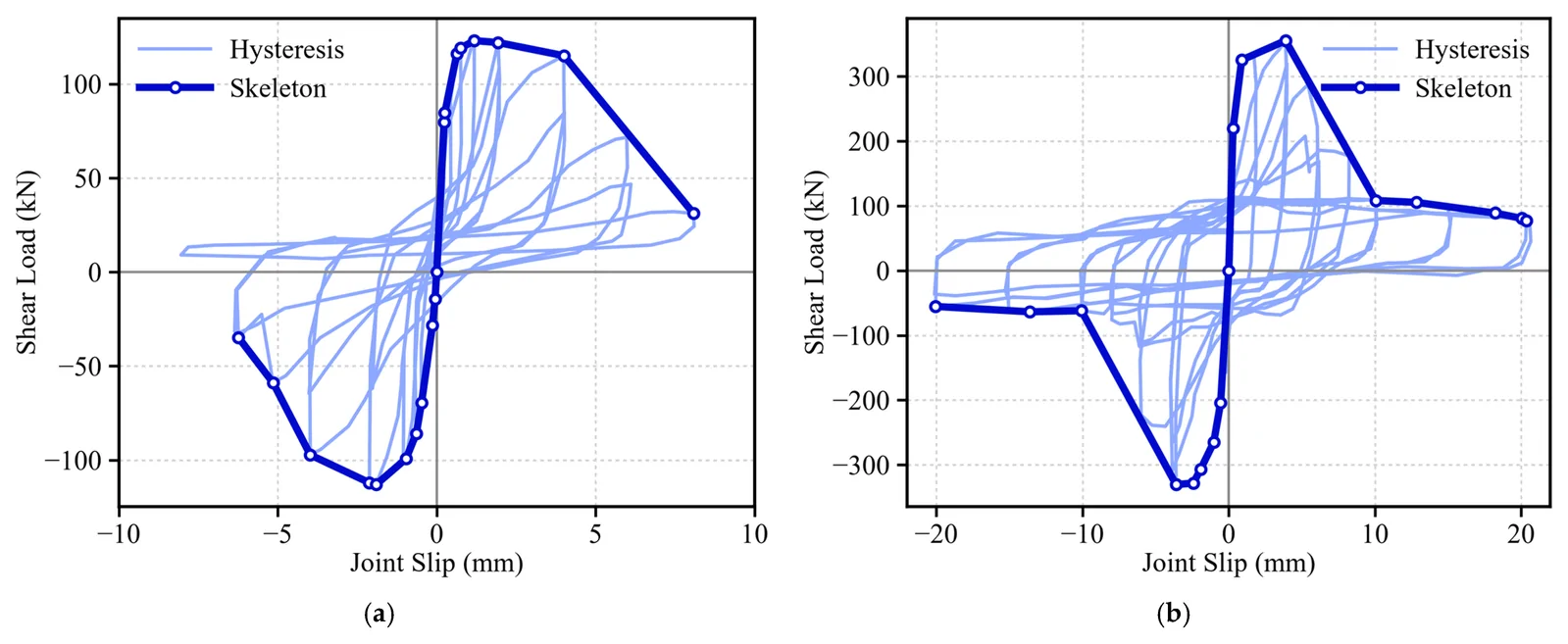
Shear load–joint slip hysteresis curves of the cyclic shear specimens: (**a**) TS16; (**b**) CS16.

Figure 7 shows the hysteretic shear load–joint slip responses of the cyclic shear specimens. The hysteretic responses were consistent with the observed damage characteristics. The tension–shear specimens showed narrow and pinched hysteretic loops, which corresponded to the two-sided interface cracking and pronounced joint slip observed during testing. In contrast, the compression–shear specimens showed fuller hysteretic loops, especially before severe anchor-ring damage occurred. This difference suggests that normal compression helped maintain interface contact during load reversal and reduced the degree of slip-dominated cyclic response.

**Figure 8:**
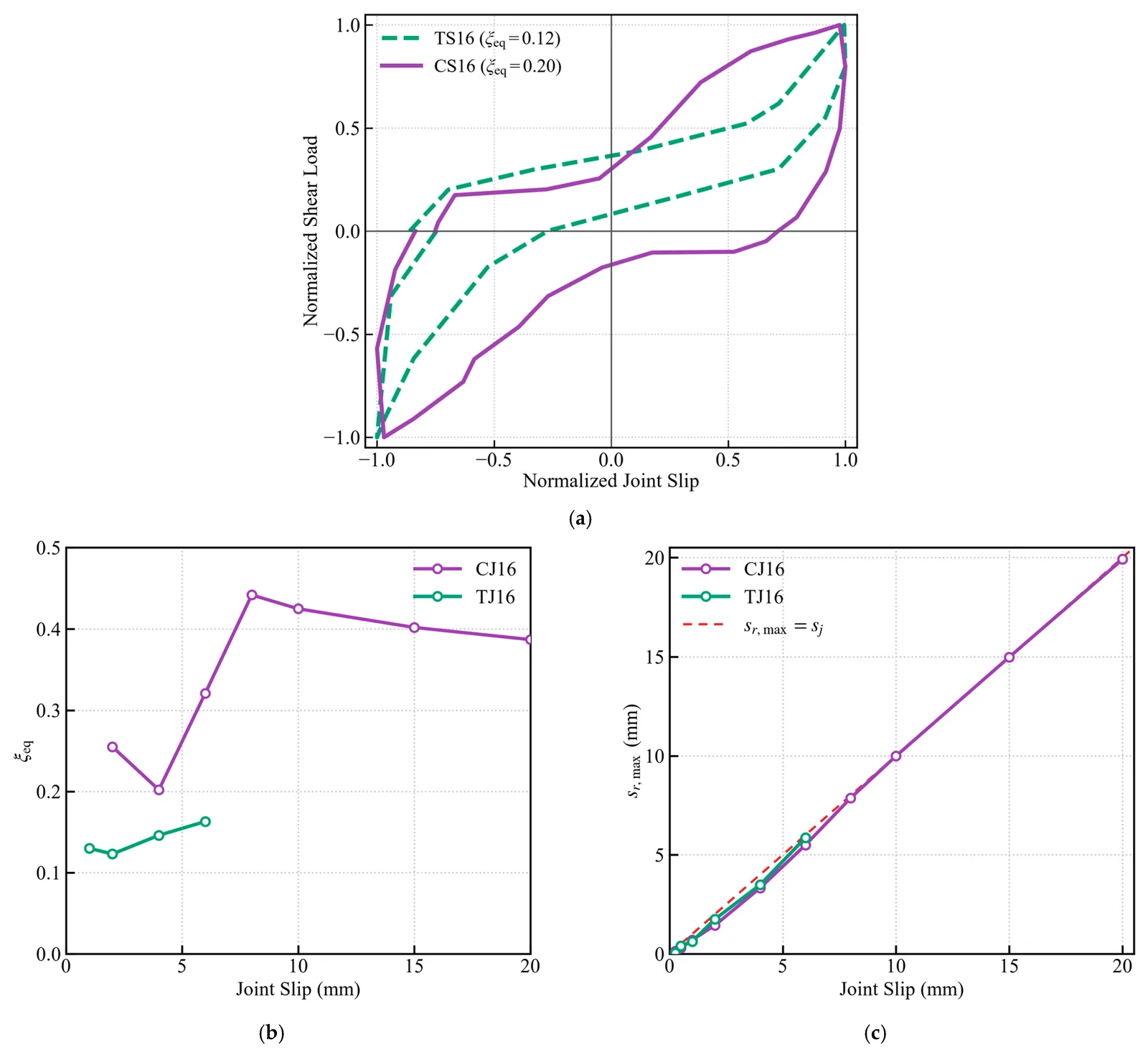
Cyclic response characteristics of the joint specimens: (**a**) normalized hysteretic loops at the peak-resistance loading levels; (**b**) equivalent viscous damping coefficients; and (**c**) maximum residual joint slips.

Figure 8 further quantifies the cyclic response characteristics of the two specimens. The equivalent viscous damping coefficients for each hysteretic loop was determined as [45,46]:

(1) $$\xi_{\mathrm{eq}}\;=\;\frac{E_{D}}{2\pi (E_{e}^{+}+E_{e}^{-})}$$

where $E_{D}$ is the area enclosed by the hysteretic loop, calculated by trapezoidal integration of the recorded shear load–joint slip data for each cycle, and $E_{e}^{+}$ and $E_{e}^{-}$ are the equivalent elastic strain energies determined from the positive and negative peak points, respectively.

Figure 8a compares the normalized hysteretic loops at the loading levels corresponding to the peak resistance, namely joint-slip amplitudes of 2 mm for TS16 and 4 mm for CS16. These loading levels were selected because they represented the fully mobilized cyclic response of each specimen before pronounced post-peak degradation. In each loading direction, the shear load and joint slip were normalized by their corresponding directional peak values. The normalized loops were used only to compare hysteretic shapes, whereas the equivalent viscous damping coefficients were calculated from the original unnormalized data. TS16 exhibited more pronounced pinching, with an equivalent viscous damping coefficient of 0.12, whereas CS16 showed a fuller loop with a corresponding coefficient of 0.20. As shown in Figure 8b, the equivalent viscous damping coefficient of CS16 remained consistently higher than that of TS16 and increased more rapidly beyond the peak-resistance loading level. These results indicate that normal compression improved hysteretic fullness and normalized energy dissipation, whereas normal tension promoted a more slip-dominated cyclic response. A similar influence of normal stress has been reported for grouted vertical joints [37], where normal tension promoted interface opening, cracking, and sliding, whereas normal compression helped maintain interface contact.

Figure 8c presents the relationship between the maximum residual joint slip and the imposed joint-slip amplitude. The residual joint slip at each loading level was characterized by the maximum absolute zero-force intercept of the unloading branches:

*s_r_*_,max_ = max (|*s_r_*^+^|, |*s_r_*^−^|) (2)

where *s_r_*^+^ and *s_r_*^−^ are the residual slips obtained during unloading from the positive and negative loading directions, respectively. For both specimens, the residual slip increased approximately in proportion to the imposed slip and represented a substantial part of the deformation at the larger loading levels. This behavior was consistent with the steep unloading branches, along which the shear load decreased rapidly to zero with only limited reverse slip. Consequently, only a small proportion of the imposed deformation was recovered during unloading, leaving substantial irreversible joint slip.

**Figure 9:**
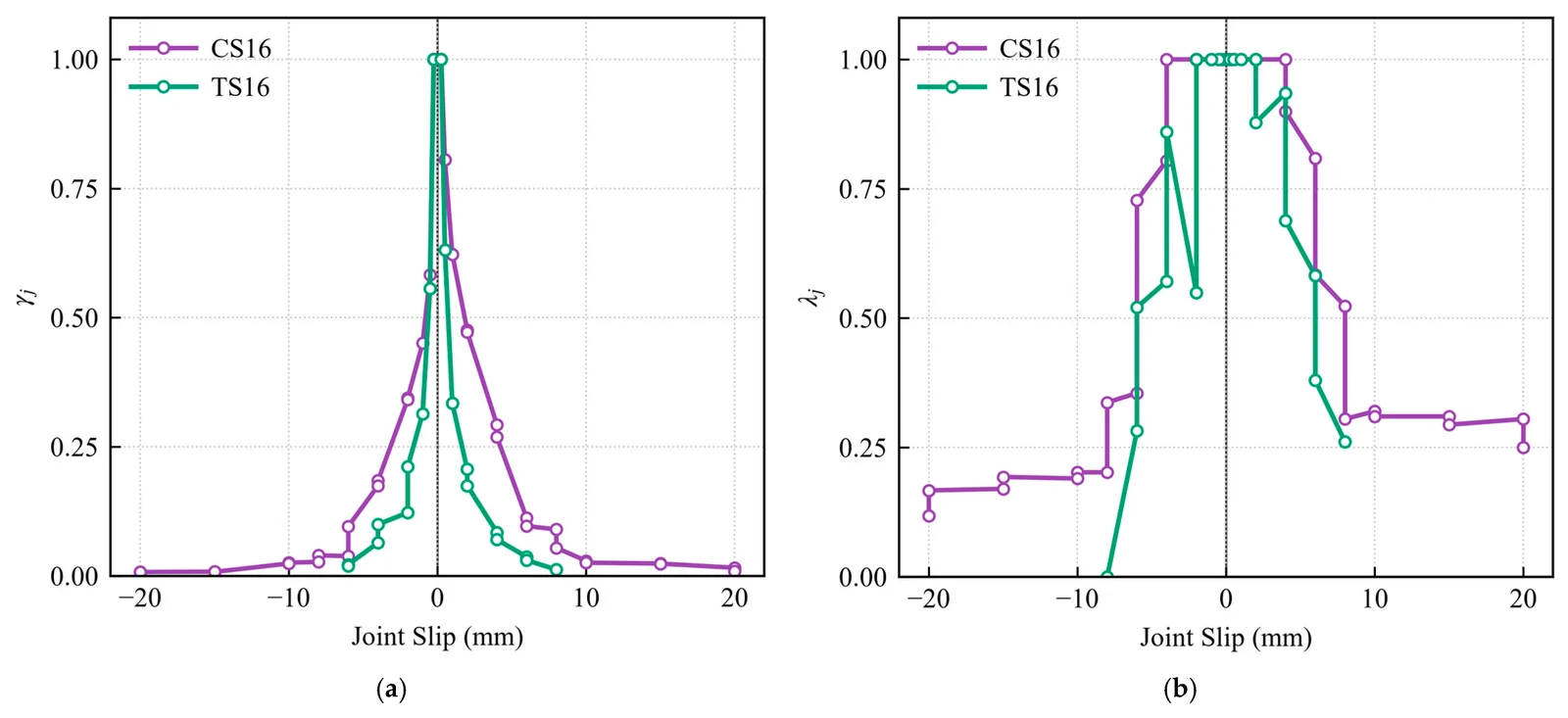
Cyclic degradation characteristics of the joint specimens: (**a**) secant stiffness degradation coefficients; and (**b**) post-peak normalized strength coefficients.

Figure 9a shows the secant stiffness degradation of the two specimens. The positive and negative secant stiffnesses at the *i*-th loading cycle were calculated separately as:

*K_j_*_,*i*_^+^ = |*V_j_*_,*i*_^+^|/|*s_j_*_,*i*_^+^|, *K_j_*_,*i*_^−^ = |*V_j_*_,*i*_^−^|/|*s_j_*_,*i*_^−^| (3)

where *V_j_*_,*i*_^+^ and *V_j_*_,*i*_^−^ are the positive and negative peak shear loads, and *s_j_*_,*i*_^+^ and *s_j_*_,*i*_^−^ are the corresponding joint slip. The stiffness degradation coefficients in the two loading directions were then defined as:

*γ_j_*_,*i*_^+^ = *K_j_*_,*i*_^+^/*K_j_*_,0_^+^, *γ_j_*_,*i*_^−^ = *K_j_*_,*i*_^−^/*K_j_*_0_^−^ (4)

where *K_j_*_,0_^+^ and *K_j_*_,0_^−^ are the initial secant stiffnesses in the corresponding loading directions. Both specimens exhibited progressive stiffness degradation with increasing joint slip. The stiffness of TS16 degraded more rapidly because normal tension promoted interface opening, bond degradation, and slip. In contrast, CS16 showed slower stiffness degradation because normal compression helped maintain interface contact. At larger joint slips, the stiffnesses of both specimens decreased to relatively low levels following substantial interface degradation and anchor-ring damage.

Figure 9b presents the post-peak normalized strength coefficients of the two specimens. For each loading direction, the peak shear load of each complete post-peak cycle was normalized by the maximum resistance attained in the corresponding direction:

*λ_j_*_,*i*_^+^ = |*V_j_*_,*i*_^+^|/|*V_j_*_,max_^+^|, *λ_j_*_,*i*_^−^ = |*V_j_*_,*i*_^−^|/|*V_j_*_,max_^−^| (5)

where *V_j_*_,*i*_^−^ and *V_j_*_,*i*_^−^ are the maximum shear resistances attained in the positive and negative loading directions, respectively. The directional peak-resistance points were assigned a normalized strength coefficient of 1.0 and taken as the starting points of the post-peak degradation curves. Only the peak-resistance points and subsequent post-peak cycles were included. Both specimens exhibited a pronounced strength reduction immediately after reaching the peak resistance. TS16 showed more rapid post-peak degradation, whereas CS16 retained a relatively stable residual strength at larger joint slips. The slower degradation of CS16 was consistent with the maintenance of interface contact and friction under normal compression.

**Figure 10:**
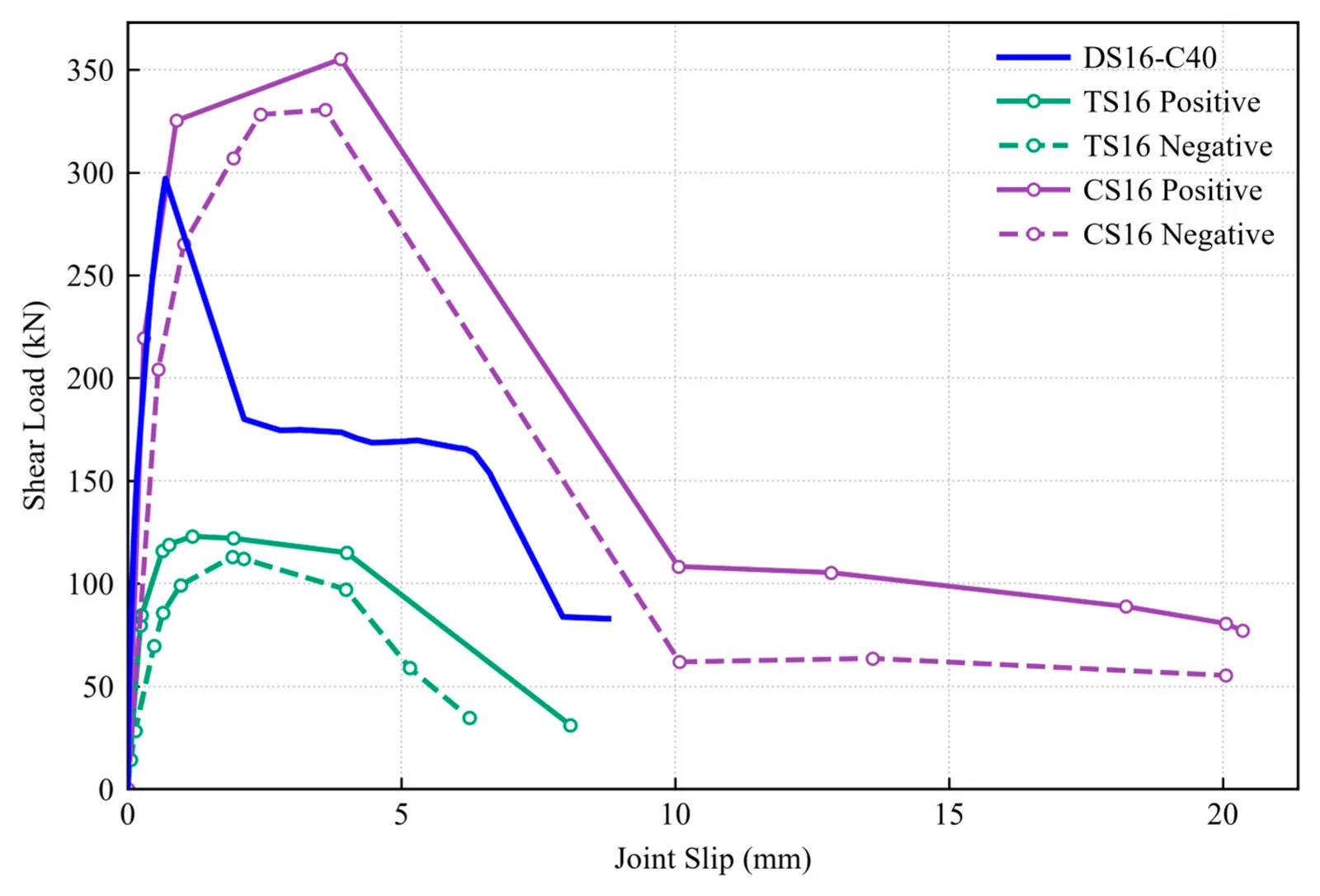
Comparison of skeleton curves of cyclic shear and direct shear specimens.

Figure 10 compares the skeleton curves of TS16 and CS16 with the monotonic shear load–joint slip curves of the corresponding direct shear specimens. For TS16, the peak resistance was much lower than that of the direct shear specimen with the same anchor-ring size. The positive and negative peak resistances of TS16 were 123 kN and 113 kN, respectively, giving an average absolute peak resistance of 118 kN. This indicates that normal tension substantially reduced the effective interface bond contribution and caused the joint to enter an anchor-ring-dominated response at a relatively low resistance level.

For CS16, the opposite trend was observed. The peak resistances were 355 kN and 330 kN in the two loading directions, giving an average absolute peak resistance of 343 kN, which was higher than that of the corresponding direct shear specimen. This confirms that normal compression enhanced interface contact and friction along the grout–panel interfaces, thereby increasing the cyclic shear resistance of the joint.

The post-peak behavior shown in Figure 10 also differed between the direct shear and cyclic shear specimens. The direct shear specimen showed a rapid resistance drop after the peak, corresponding to sudden degradation of the interface bond once major debonding occurred. By contrast, the cyclic shear specimens showed more gradual post-peak degradation. For CS16, this gradual degradation can be attributed to normal compression, which enhanced interface contact and slowed down bond degradation. For TS16, the mechanism was different. Normal tension caused early degradation of the interface bond before the peak resistance was reached. As a result, the shear-transfer mechanism had already shifted largely to anchor-ring-dominated resistance at the peak stage, leading to a relatively gradual post-peak decrease rather than a sudden drop.

The cyclic shear results show that the normal-force condition significantly affected the hysteretic response, peak resistance, and post-peak degradation of the joint. Normal tension weakened the interface bond between the grout and precast wall panels, resulting in two-sided interface cracking, pinched hysteretic loops, and much lower peak resistance. Normal compression, in contrast, enhanced interface contact and friction, leading to one-sided interface cracking, fuller hysteretic loops, higher peak resistance, and more stable residual resistance after anchor-ring fracture.

### 3.3. Shear Resistance Evaluation

The shear resistance of the joint specimens was evaluated using the expression specified in Clause 5.7.8 of GB/T 51231 [47] for precast concrete wall-to-wall joints:

*V_j_*_,c_ = 0.6*f_j_*_,*y*_*A_j_*_,*s*_ + 0.8*N_j_* (6)

where *f_j_*_,*y*_ and *A_j_*_,*s*_ denote the yield strength and effective area of the steel connectors, respectively, and *N_j_* is the normal force perpendicular to the joint, taken as positive in compression and negative in tension.

**Figure 11:**
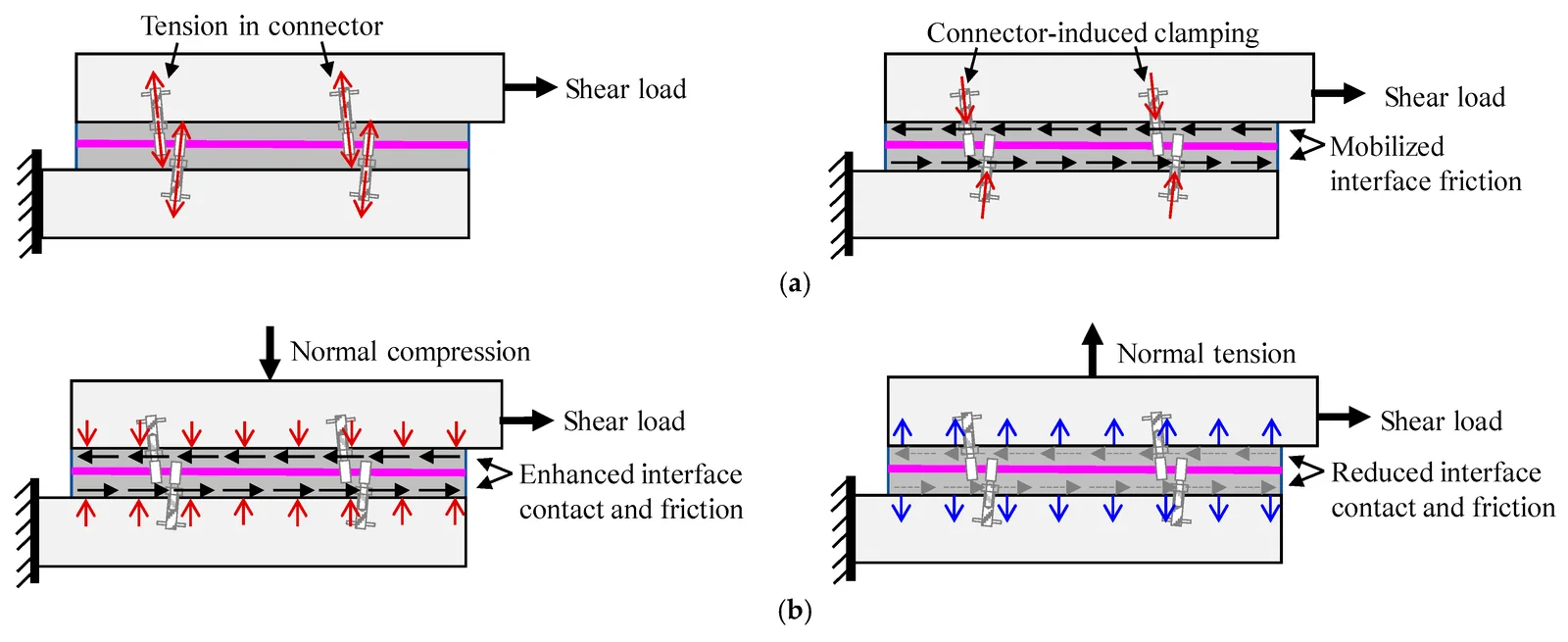
Schematic interpretation of the shear-friction terms in Equation (6): (**a**) connector contribution mobilized by joint slip; and (**b**) influence of external normal force.

This expression is an empirical shear-friction model, with the schematic interpretation of the two terms presented in Figure 11. The first term, 0.6*f_j_*_,*y*_*A_j_*_,*s*_, represents the empirical contribution of the steel connectors. As illustrated in Figure 11a, joint slip deforms the connectors and mobilizes a restraining action across the interface, thereby helping maintain interface contact and develop frictional resistance. Under this deformation, the connectors may be subjected to a combination of shear, bending, local bearing associated with dowel action, and local tension. These individual actions are not resolved separately in the code expression. Instead, their combined mechanical contribution is represented by the product *f_j_*_,*y*_*A_j_*_,*s*_ multiplied by an empirical coefficient of 0.6, which was calibrated through regression of previous tests on precast wall-to-wall joints. The second term, 0.8*N_j_*, accounts for the influence of the external normal force, with 0.8 likewise serving as an empirical coefficient. As shown in Figure 11b, normal compression enhances interface contact and friction, whereas normal tension reduces them. Comparable empirical treatments are also adopted in ACI 318 and Eurocode 2, as introduced and compared below.

For the proposed joint, connector yield strength, connector area, and normal force in the original expression were mapped to *f_j_*_,*y*_, *A_j_*_,*s*_ and *N_j_*, respectively. Specifically, *f_j_*_,*y*_ was taken as the measured yield strength of the anchor-ring shank material, and the anchor-ring shanks were idealized as the discrete steel connectors participating in the equivalent shear-friction mechanism. Because the two opposed anchor rings in each connection pair formed a single force-transfer path through the inserted vertical bar, one effective shank cross-section was counted for each connection pair. Each joint-level specimen contained two connection pairs; therefore, *A_j_*_,*s*_ was taken as the sum of two effective shank cross-sectional areas, calculated using the nominal shank diameter. The normal force *N_j_* was taken as the applied normal force perpendicular to the joint using the same sign convention as the original expression.

To examine the applicability and conservatism of this code-based expression for steel-anchor-ring grouted vertical joints, the measured-to-calculated resistance ratio *η_j_* was calculated as:

*η_j_* = *V*_max_/*V_j_*_,c_ (7)

where *V*_max_ is the measured peak shear resistance. The values of *V_j_*_,c_, *V*_max_, and *η_j_* are summarized in Table 4.

For the direct shear specimens, the measured-to-calculated resistance ratios *η_j_* ranged from 1.97 to 2.70. The reference specimen DS16-C40 had an *η_j_* value of 2.70, while DS20-C40 and DS16-C30 had values of 1.97 and 2.34, respectively. For the tension–shear specimen TS16, the *η_j_* values were 1.31 and 1.20 in the two loading directions, respectively. For the compression–shear specimen CS16, the ratios increased to 2.50 and 2.32. The lower ratios obtained for TS16 were consistent with the observed interface opening and bond degradation under normal tension. All measured-to-calculated ratios were greater than unity, indicating that the code-based expression underpredicted the measured peak resistance of the tested specimens.

To further evaluate the applicability of the anchor-ring-based expression, representative interface-shear models from BS 8110 [48], ACI 318 [49], and Eurocode 2 [50] were also used for comparison. These models were originally developed for shear transfer across concrete-to-concrete interfaces or construction joints, and therefore they were used here as comparative benchmarks.

For BS 8110, the shear resistance was calculated as:

*V_j_*_,BS_ = 0.6*k*_s_*f_j_*_,*y*_*A_j_*_,*s*_ (8)

where *k*_s_ is the surface-condition factor, taken as 0.7 and 1.4 for smooth and rough joints, respectively. Since the proposed joint had smooth grout–panel interfaces, *k*_s_ = 0.7 was adopted.

For ACI 318, the shear resistance was evaluated as:

*V_j_*_,ACI_ = *μf_j_*_,*y*_*A_j_*_,*s*_ ≤ [min(0.2*f*_c_, 5.52)*A_b_*] (9)

where *μ* is the coefficient of friction along the assumed shear plane, and *A_b_* is the bonded area between the grout and the precast wall panels. For smooth interfaces, *μ* = 0.6 was adopted.

For Eurocode 2, the shear resistance was evaluated as:

*V_j_*_,EC2_ = *cf*_t_*A_b_* +*μN_j_* + *f_j_*_,*y*_*A_j_*_,*s*_ (*μ* sin *a* + cos *a*) (10)

where *c* is the cohesion coefficient representing the bond contribution of the interface, and *μ* is the friction coefficient representing the shear-friction contribution associated with the normal force and the reinforcement crossing the interface. Both *c* and *μ* depend on the surface roughness and were taken as 0.2 and 0.6, respectively, for smooth interfaces. α is the inclination angle between the reinforcement and the interface, taking as 90° for the proposed joint.

These models share a common shear-friction basis, in which the steel elements crossing the shear plane contribute to interface shear resistance through reinforcement-related shear-friction action. For smooth interfaces, the GB/T 51231, ACI 318, and Eurocode 2 models give comparable steel-related coefficients of 0.6, whereas BS 8110 gives a lower effective coefficient of 0.42 because of the additional surface-condition factor. The main difference lies in the treatment of normal force and interface bond. BS 8110 and ACI 318 do not include the applied normal force, whereas the GB/T 51231 and Eurocode 2 models include a normal-force term. The coefficient for this term is 0.8 in GB/T 51231 and 0.6 in Eurocode 2 for smooth interfaces. In addition, only Eurocode 2 includes a cohesion term to account for interface bond.

**Figure 12:**
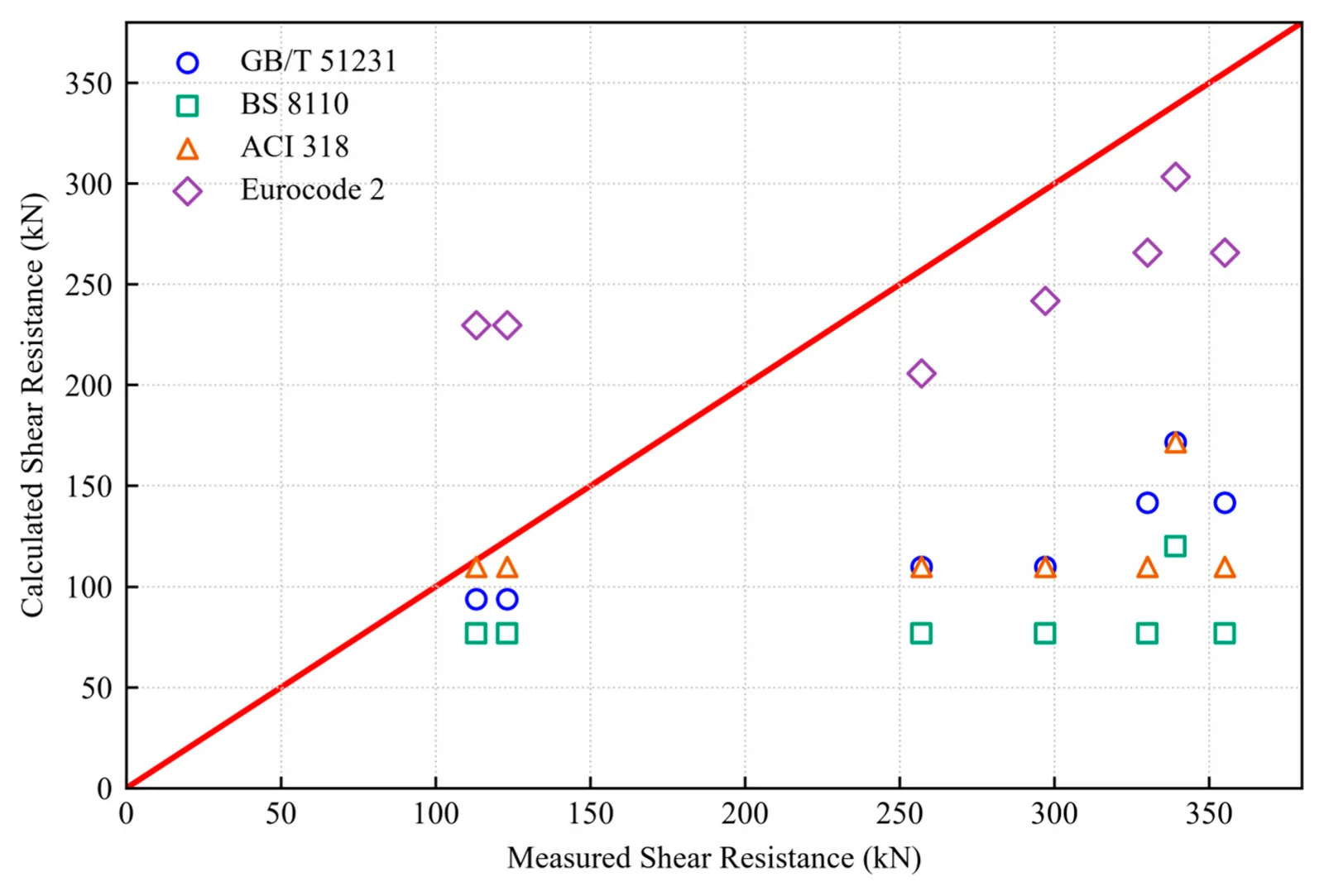
Comparison between measured and code-calculated shear resistances.

Figure 12 compares the measured peak shear resistance with the calculated resistances from the GB/T 51231, BS 8110, ACI 318, and Eurocode 2. The diagonal line represents equality between the calculated and measured values; points below the line indicate underprediction, whereas points above the line indicate overprediction. Eurocode 2 gave the highest calculated resistances because it includes an explicit cohesion term for interface bond. This improved the prediction for the direct shear and compression–shear specimens, but led to overestimation for the tension–shear specimens, where normal tension caused interface opening and early bond degradation. ACI 318 generally underpredicted the measured values, although its predictions for the tension–shear specimen were close to the measured resistance because the adverse effect of normal tension is not explicitly included. BS 8110 produced the lowest calculated values because its steel-related contribution for smooth interfaces is further reduced by the surface-condition factor. The GB/T 51231 expression underpredicted all measured peak resistances because it excluded the bond contribution, while accounting for the effect of normal force.

## 4. Wall-Level Cyclic Tests

### 4.1. Design of Wall Specimens and Vertical Joint

Two precast shear wall specimens with steel-anchor-ring grouted vertical joints were designed, namely W1mid and W2off. The specimens were rectangular wall panels with a thickness of 200 mm. W1mid had a wall length *L_w_* of 1000 mm and a shear span ratio of 2.80, whereas W2off had a wall length *L_w_* of 2000 mm and a shear span ratio of 1.40. The wall height was 2800 mm, and the vertical joint length *L_j_* was also 2800 mm for both specimens.

**Figure 13:**
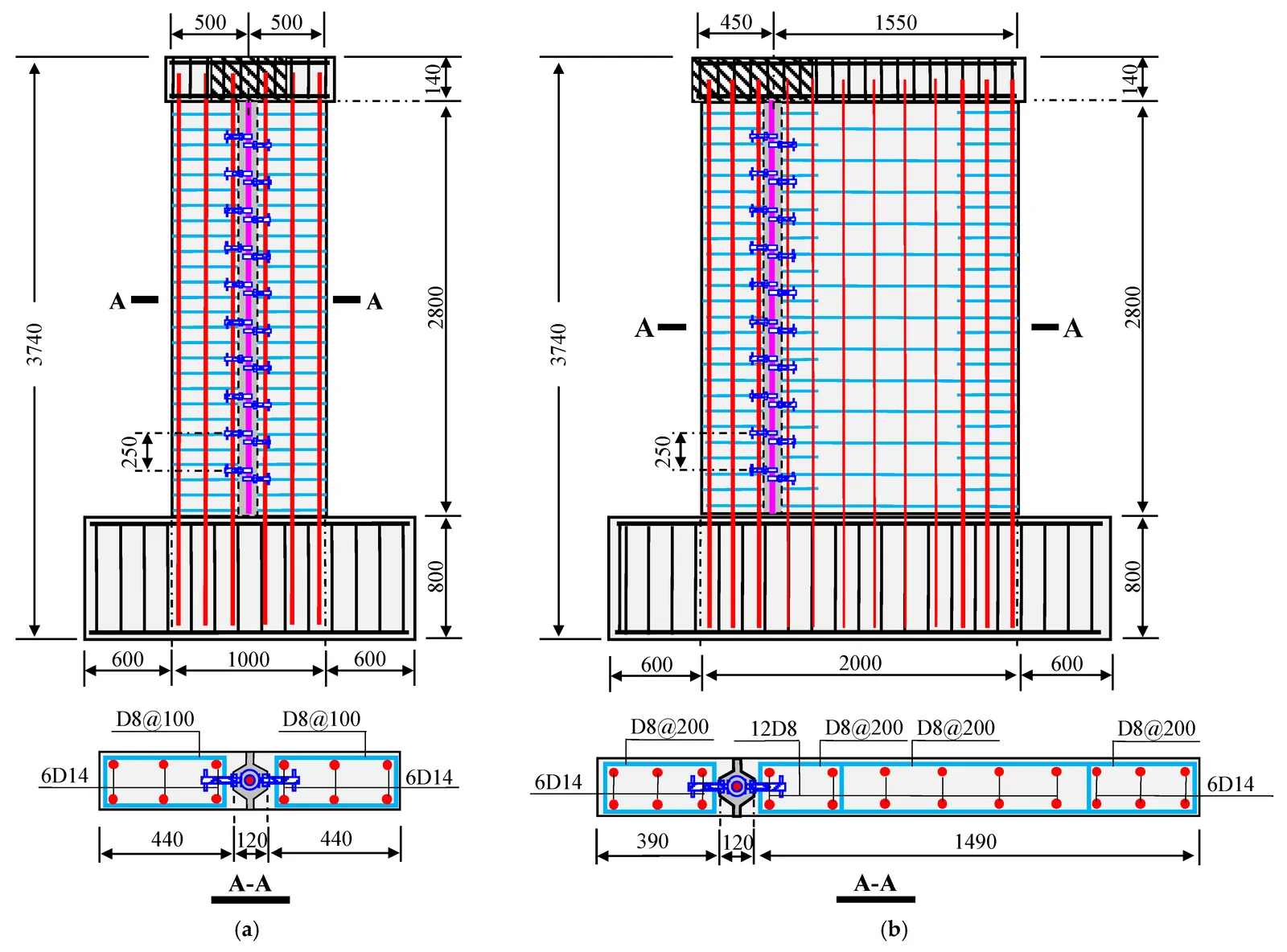
Details and reinforcement layouts of wall specimens W1mid and W2off: (**a**) W1mid; (**b**) W2off.

The two specimens had different vertical-joint locations, as shown in Figure 13. The parameter *d_j_* denotes the distance from the vertical joint to the nearest wall edge, measured along the wall length. In W1mid, the vertical joint was placed at the wall mid-length, with *d_j_* = 500 mm. In W2off, the vertical joint was offset from the wall edge, with *d_j_* = 450 mm. Both vertical joints adopted M20 steel anchor rings arranged at a vertical spacing of 250 mm, with 10 pairs of anchor rings provided along the joint.

The wall sections were designed using C40 concrete and HRB400 reinforcement. According to the code [51], the design axial compressive and tensile strengths of C40 concrete are *f*_c_ = 26.8 MPa and *f*_t_ = 2.39 MPa, respectively. The reinforcement details are shown in Figure 13. The wall web was reinforced with D8 distributed bars at a spacing of 200 mm in both vertical and horizontal directions. Boundary elements were provided at the wall edges. A constant axial compression of 1100 kN was applied to both specimens during testing. Based on *f*_c_, the nominal axial compression ratio was 0.20 for W1mid and 0.10 for W2off.

The design wall shear force was determined from the flexural resistance of the wall section. Sectional equilibrium under axial compression was used to calculate the flexural resistance. For W1mid, the calculated flexural resistance was 728 kN·m, corresponding to a design wall shear force of 260 kN. For W2off, the calculated flexural resistance was 1487 kN·m, corresponding to a design wall shear force of 531 kN.

The shear demand of the vertical joint was estimated using a linear-elastic shell model established in SAP2000. The wall panels were modeled using four-node thin-shell elements incorporating in-plane membrane and out-of-plane bending behavior, with the actual wall thickness of 200 mm. Concrete was represented as a homogeneous and isotropic linear-elastic material, with an elastic modulus of 30 GPa and a Poisson’s ratio of 0.20. To reproduce the test boundary condition, all nodes along the wall base were fully restrained in translation and rotation. The axial force was applied uniformly along the top edge of the wall as a line load. The horizontal force, equal to the selected design wall shear force, was distributed uniformly among the nodes at the loading level to represent the load distribution provided by the loading beam. The wall panels on the two sides of the nominal vertical-joint line were modeled with compatible nodes, without introducing interface opening, slip, or nonlinear joint behavior.

Section cuts were defined directly along the nominal vertical-joint lines, and the integrated vertical shear force across each cut was taken as the elastic joint-demand estimate *V_j_*_,*d*_ at the selected design wall shear force. Because the model imposed displacement compatibility along the nominal joint and did not include interface opening, slip, or nonlinear joint behavior, *V_j_*_,*d*_ should not be interpreted as the actual nonlinear joint force at the ultimate wall response. The resultant force perpendicular to each section cut was also extracted as the joint-normal force *N_j_*, taken as positive in compression and negative in tension. A mid-joint reference case was also considered by defining an additional section cut at the wall mid-length in the same model used for W2off. This comparison therefore isolated the analytical influence of joint location on the transferred shear demand.

**Figure 14:**
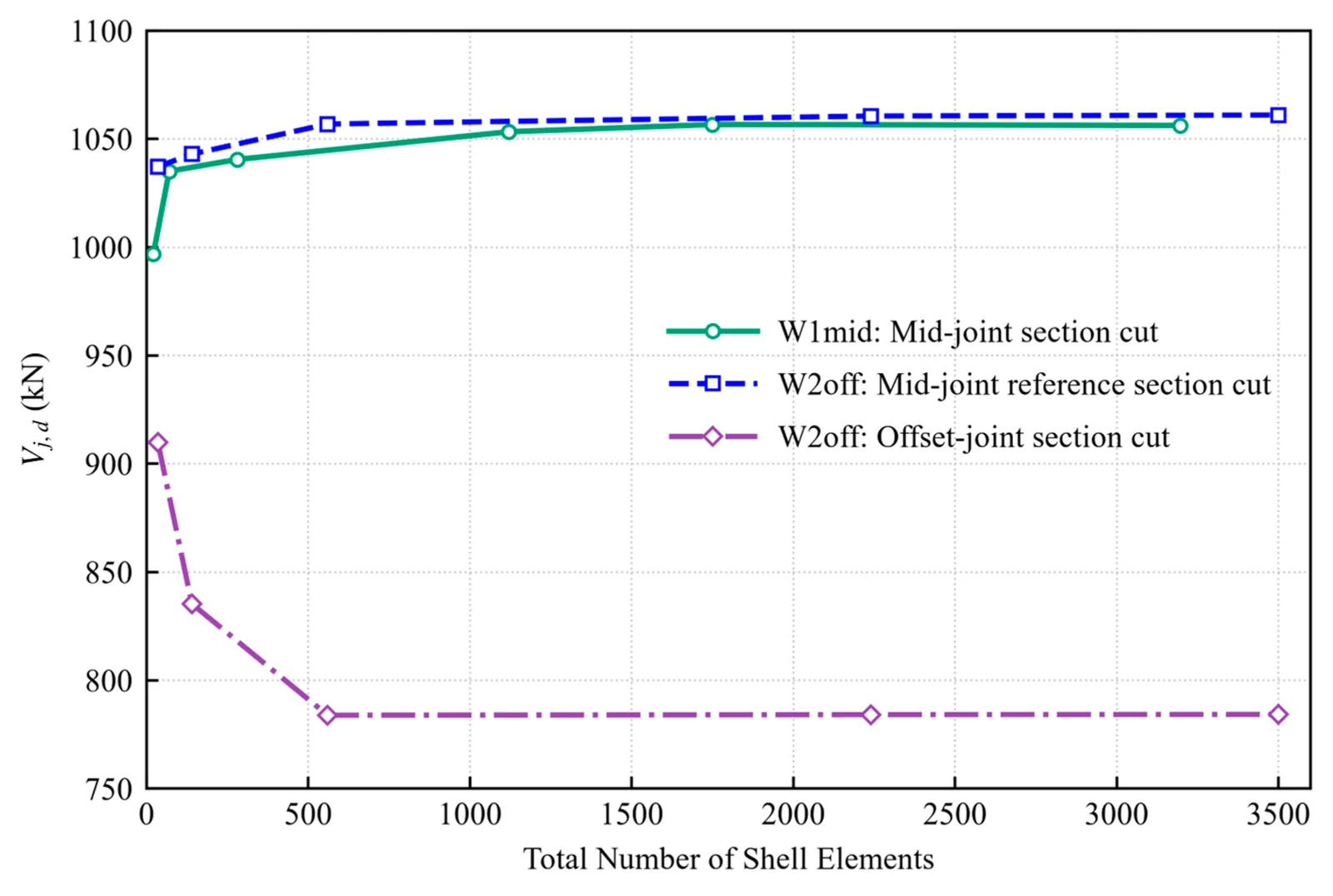
Mesh Sensitivity Analysis of Elastic Joint Demand.

Mesh sensitivity was examined using nominal element sizes of 400, 200, 100, 50, and 40 mm. For the smaller W1mid model, an additional mesh size of 30 mm was considered. Figure 14 presents the calculated elastic joint-demand estimate against the total number of shell elements. For the mid-joint section cuts of W1mid and the W2off reference case, *V_j_*_,*d*_ increased gradually with mesh refinement and approached stable values. For the offset-joint section cut of W2off, *V_j_*_,*d*_ decreased more noticeably with the initial mesh refinement and then similarly converged. Further refinement beyond a nominal element size of 50 mm produced only minor changes in all three section-cut results. Therefore, a nominal mesh size of 50 mm was adopted for both wall models in the subsequent analyses.

**Figure 15:**
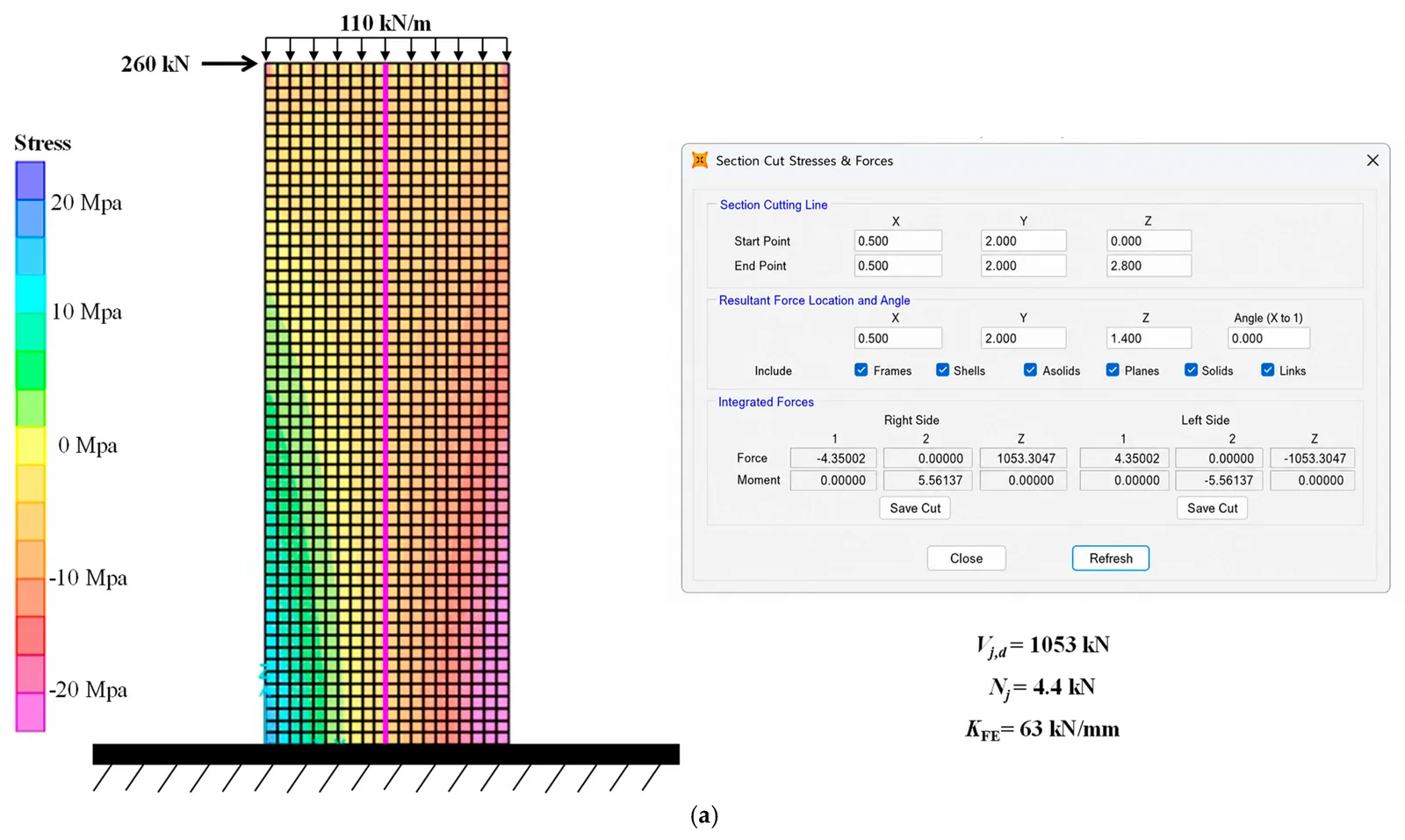

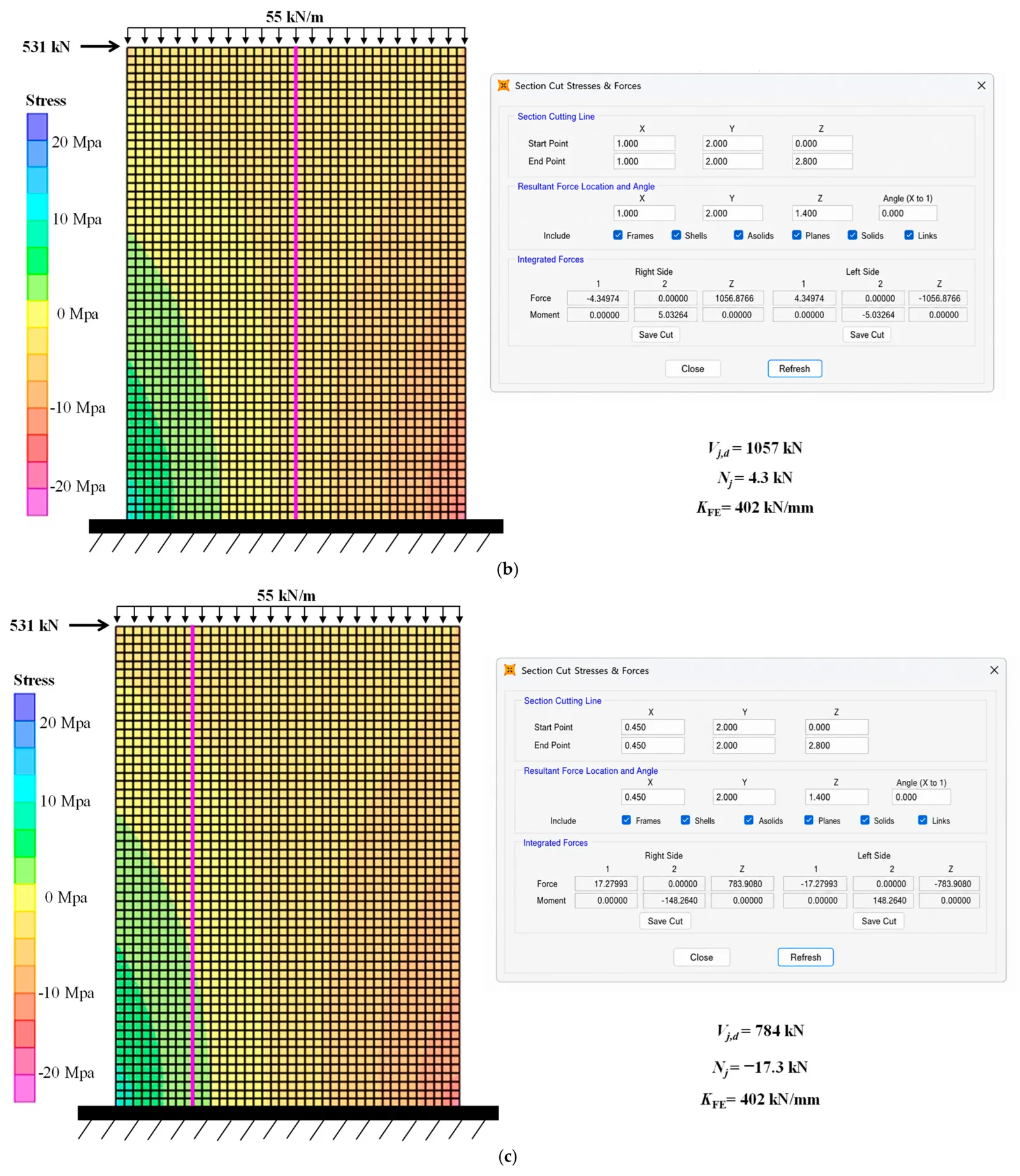
Elastic shell models and section-cut definitions for evaluating vertical-joint shear demand: (**a**) W1mid, 1000 mm wall with a mid-length vertical joint; (**b**) 2000 mm wall with a mid-length vertical joint; (**c**) W2off, 2000 mm wall with an offset vertical joint.

The comparison between W1mid and the 2000 mm mid-joint reference case, as shown in Figure 15a,b, indicates that the shear span ratio had a marked influence on the elastic joint shear demand. Both cases considered a vertical joint at the wall mid-length. Although W1mid had a lower design wall shear force, its larger shear span ratio resulted in a mid-joint demand estimate of *V_j_*_,*d*_ = 1053 kN, which was close to the value of *V_j_*_,*d*_ = 1057 kN obtained for the 2000 mm mid-joint reference case. This comparison indicates that the shear demand at a mid-length vertical joint is not determined solely by the global wall shear force but is also strongly influenced by the wall shear span ratio.

The influence of vertical-joint location can be further clarified by comparing the mid-joint and offset-joint section cuts within the same 2000 mm wall model, as shown in Figure 15b,c. The wall geometry, shear span ratio, boundary conditions, and applied loads were identical, and only the section-cut location differed. The mid-joint reference cut gave an elastic joint-demand estimate of *V_j_*_,*d*_ = 1057 kN, whereas the offset cut corresponding to W2off, located at *d_j_* = 450 mm, gave *V_j_*_,*d*_ = 784 kN. This represents a reduction of 25.8%, indicating that locating the vertical joint away from the wall mid-length can reduce the elastic shear demand transferred across the joint.

The section-cut analysis also showed that normal forces developed across the vertical joints under the combined vertical and lateral loads. Owing to symmetry, the lateral load did not produce normal resultant across the mid-joint. The applied axial load produced only a small compressive resultant of 4.4 kN, attributable to the restraint of transverse deformation at the fixed wall base. The mid-joint therefore remained in compression throughout the analysis. By contrast, the offset-joint cut in W2off developed a varying normal resultant under lateral loading because of nonuniform in-plane stress transfer, ranging from 24.0 kN in compression to 17.3 kN in tension. Based on *f*_t_, the normal tensile stress ratio was 0.013, lower than the value of 0.03 applied to Specimen TS16. The analysis therefore indicates that the wall-to-wall vertical joints were subjected to low normal stress demands under the considered loading conditions [29,30]. In addition, the linear-elastic SAP2000 models predicted lateral stiffnesses *K*_FE_ of 63 kN/mm for W1mid and 402 kN/mm for W2off. These predictions are compared with the measured elastic-range responses in Section 4.5.

The joint shear demands were checked against the code-based joint resistance *V_j_*_,*c*_. Both wall specimens used M20 steel anchor rings arranged at a spacing of 250 mm, resulting in *V_j_*_,*c*_ = 858 kN. In addition, the measured-to-calculated resistance ratio *η_j_* = 1.97, obtained from the corresponding direct shear specimen DS20-C40, was applied to the code resistance, giving an equivalent test-based reference resistance of *η_j_V_j_*_,*c*_ = 1690 kN for comparison with the wall-level joint demands. The design parameters, joint shear demands, and resistance check results are summarized in Table 5.

The results show that W2off had a calculated joint demand below *V_j_*_,*c*_, whereas the joint shear demand of W1mid exceeded *V_j_*_,*c*_ but remained below the equivalent test-based reference resistance. Therefore, the two tested wall specimens covered different vertical-joint demand levels: W2off represented a configuration with a calculated joint demand below the code-based reference value, whereas W1mid represented a higher-demand configuration exceeding the code-based resistance but remaining below the test-based reference resistance.

### 4.2. Material Properties for Wall-Level Tests

Material tests were conducted to obtain the mechanical properties of the concrete, grout, steel anchor rings, and reinforcement used in the wall specimens. Three 150 mm × 150 mm × 300 mm prismatic specimens and three 150 mm cubic specimens were cast from the same concrete batch as the wall specimens, cured under standard conditions, and tested at 28 days in accordance with GB/T 50081-2019 [42]. The mean axial compressive strength and splitting tensile strength of the nominal C40 concrete were 27.0 MPa and 2.6 MPa, respectively. The coefficient of variation in the measured axial compressive strength was 11%. The high-strength cementitious grout and steel anchor rings were obtained from the same batches as those used in the joint-level specimens. The mean compressive and flexural strengths of the grout were 66.1 MPa and 7.1 MPa, respectively.

Tensile tests were also conducted on three representative specimens of each reinforcing-bar diameter at room temperature in accordance with GB/T 228.1-2021 [44]. The coefficients of variation in the measured yield and ultimate tensile strengths were 0.58% and 0.80%, respectively, for the D8 bars, and 0.48% and 0.34%, respectively, for the D12 bars. The mean yield strain, yield strength, and ultimate tensile strength of the steel components are summarized in Table 6.

### 4.3. Test Setup and Loading Protocol

**Figure 16:**
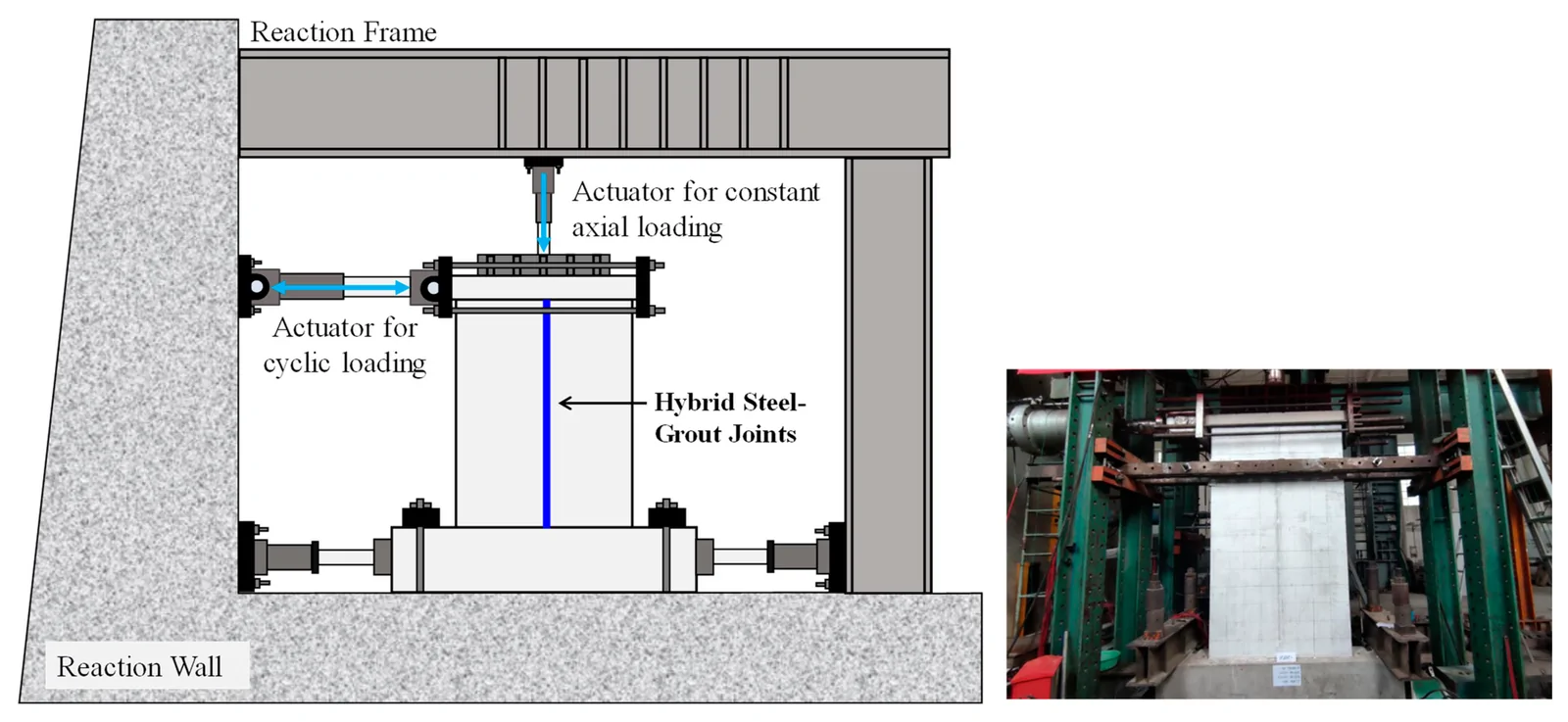
Test setup for wall specimens.

Figure 16 shows the test setup for the wall specimens. Each specimen was tested as a cantilever wall. The foundation beam was rigidly anchored to the strong floor to restrain sliding and uplift. A constant axial compression was applied to the top loading beam using vertical hydraulic actuator, while reversed cyclic lateral loading was imposed by an electro-hydraulic actuator connected to the reaction wall. Out-of-plane restraints were arranged on both sides of the specimen to prevent lateral instability without restricting the in-plane deformation of the wall. The lateral load, axial load, and top lateral displacement were recorded during the test. The wall-top lateral displacement was measured at the loading level and was used to construct the global lateral load–displacement responses.

The specimens were tested under quasi-static cyclic loading. The target axial compression was first applied and then maintained approximately constant throughout the lateral loading process. Before yielding, the lateral loading was controlled by force, with the load increased in increments of 50 kN. After yielding, the loading mode was switched to displacement control. The target displacement started from 5 mm and was increased in increments of 2.5 mm until reaching 20 mm. Thereafter, the displacement increment was increased to 5 mm. The test was terminated when the lateral resistance dropped below 85% of the peak resistance.

### 4.4. Damage Patterns of Wall Specimens

**Figure 17:**
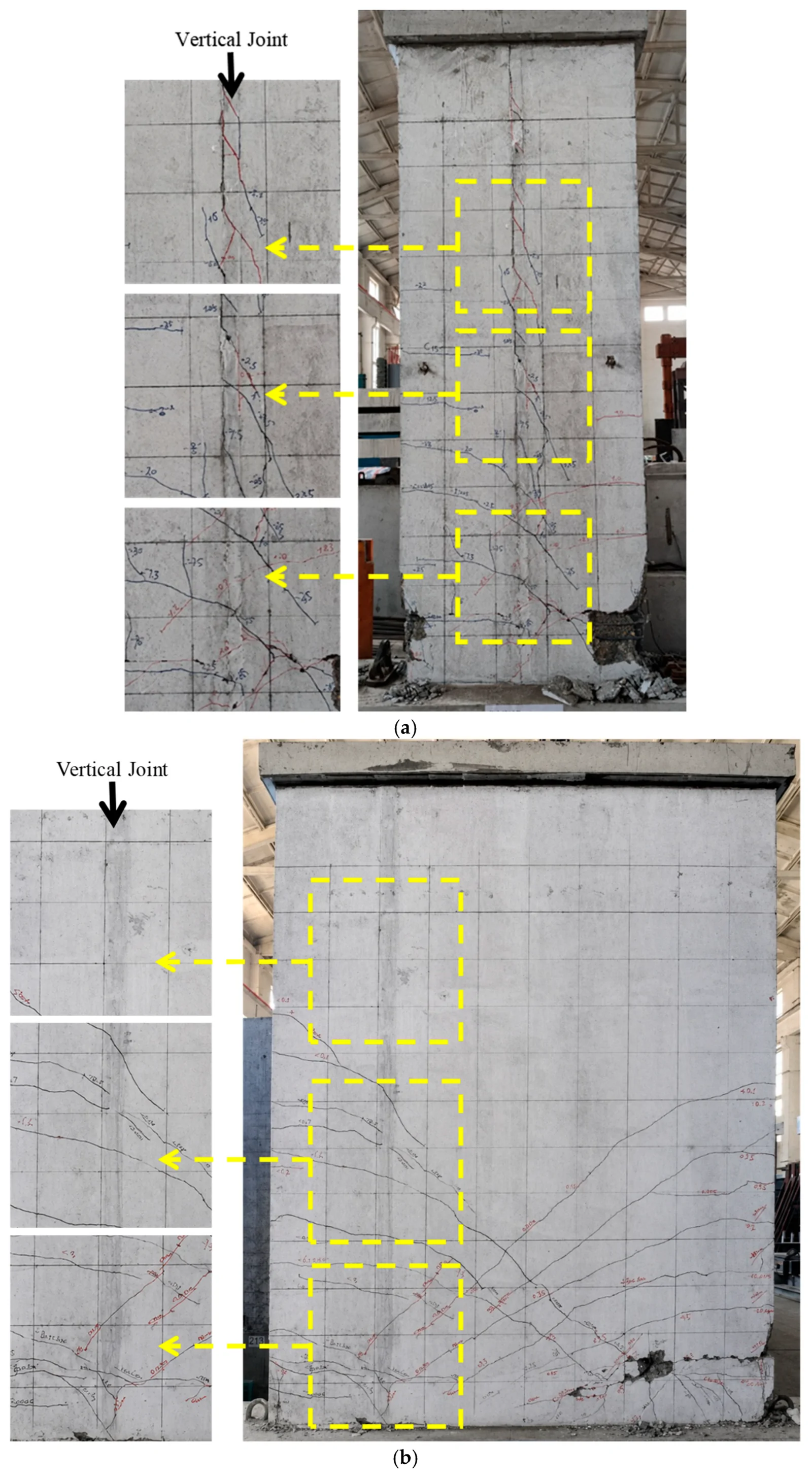
Failure modes and vertical-joint damage details of wall specimens: (**a**) W1mid; (**b**) W2off.

Figure 17 shows the failure modes and vertical-joint damage details of the wall specimens. Both specimens exhibited flexure-dominated damage, with the main damage concentrated in the plastic hinge region at the wall base. The typical damage process included flexural cracking near the wall base, crack propagation toward the wall web, local cracking along or across the vertical joint, concrete spalling and crushing at the wall corners, and final strength degradation associated with the development of the bottom plastic hinge. No severe debonding of the vertical joint or fracture of the steel-anchor-ring connection was observed during the tests.

For specimen W1mid, no visible damage was observed after axial compression. During force-controlled loading, the first horizontal flexural crack appeared near the wall base at 200 kN, and additional cracks developed and extended toward the wall web at 250 kN. After this load level, the boundary longitudinal reinforcement approached the yield strain, and the loading protocol was switched to displacement control. During displacement-controlled loading, the flexural cracks propagated and widened rapidly. At a lateral displacement of 10 mm, visible cracking appeared along the vertical joint. At 15 mm, several inclined cracks developed near the joint; some extended along the joint interface, whereas others crossed the joint and propagated into the adjacent precast wall panel, indicating that the vertical joint still transferred shear between the two wall segments. With increasing displacement, damage became concentrated near the wall base, accompanied by corner spalling, concrete crushing, and local exposure of transverse reinforcement. The test was terminated at 45 mm when the lateral resistance decreased to 85% of the peak value. Although W1mid developed visible joint-related cracking, the cracks did not propagate through the full joint height, and no severe joint separation occurred.

For specimen W2off, no visible damage was observed after axial compression or during the early force-controlled loading stage. The specimen remained almost intact before 400 kN, when a horizontal flexural crack appeared near the wall base. At 500 kN, several horizontal cracks extended toward the wall web, and the boundary longitudinal reinforcement approached the yield strain; the loading protocol was then switched to displacement control. During subsequent loading, the existing cracks continued to propagate, but the offset vertical joint showed much less damage than that of W1mid. Some inclined cracks propagated toward the offset joint and crossed into the adjacent precast wall panel, indicating continued shear transfer across the joint. However, no obvious interface cracking or severe joint damage developed. At 45 mm, corner spalling, partial concrete crushing, and local buckling of vertical reinforcement were observed. The test was terminated when the negative lateral resistance decreased to 85% of the peak value. Overall, W2off exhibited wall-base flexural damage with limited joint-related cracking, consistent with its lower calculated joint shear demand.

The damage observations indicate that the steel-anchor-ring grouted vertical joints did not govern the final failure of the wall specimens. Both specimens ultimately developed flexural damage at the wall base, while no severe joint separation or fracture of the steel-anchor-ring connections was observed. W1mid developed more visible joint-related cracking than W2off, which was consistent with the difference in their calculated joint shear demands.

### 4.5. Hysteretic Response

**Figure 18:**
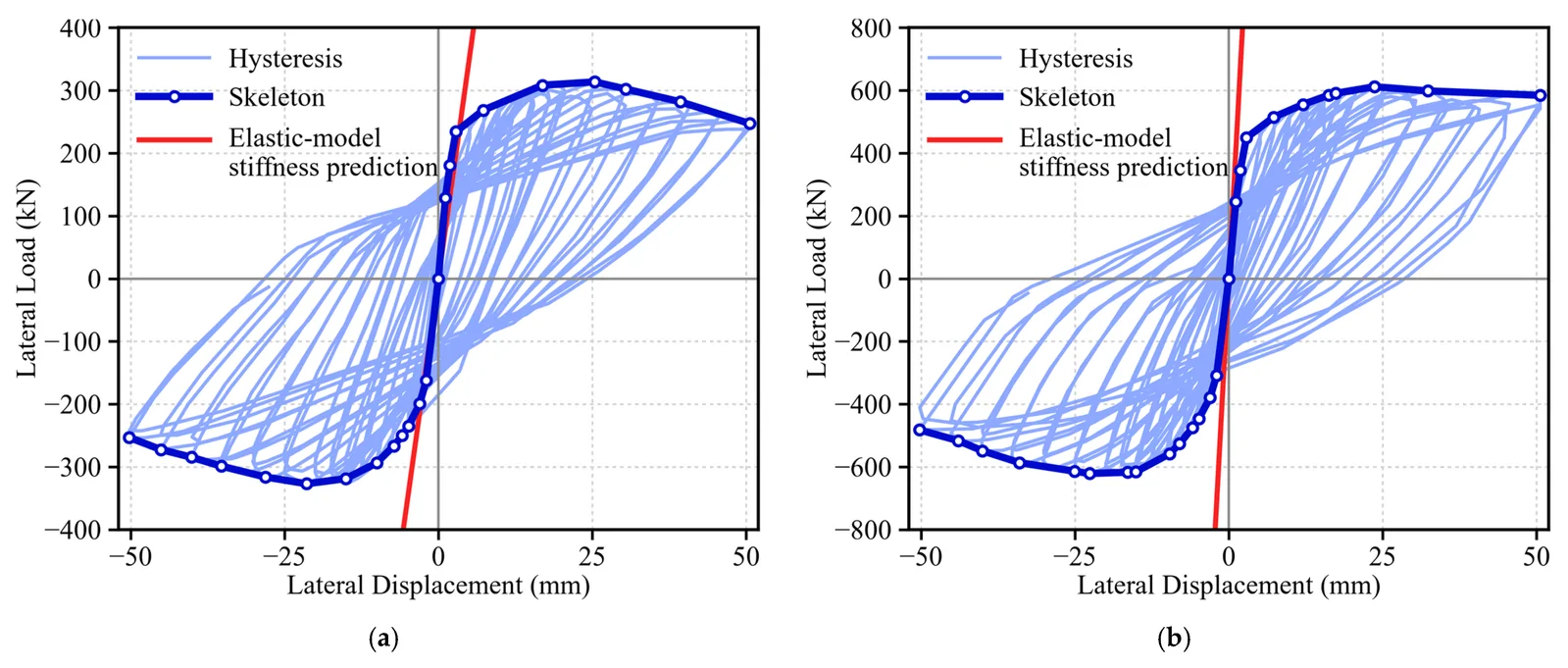
Hysteretic responses of wall specimens: (**a**) W1mid; (**b**) W2off.

Figure 18 shows the hysteretic lateral load–displacement responses of the wall specimens. Both specimens exhibited relatively full hysteretic loops without obvious slip-induced pinching, indicating that the steel-anchor-ring grouted vertical joints maintained stable shear transfer during cyclic loading. The elastic-model predictions generally captured the initial slopes of the measured responses. The relative differences between the predicted and measured initial stiffnesses were −22.8% for W1mid and 35.5% for W2off, corresponding to an underestimation for W1mid and an overestimation for W2off. Specimen W1mid reached peak lateral resistances of 313.6 kN and 327.0 kN in the positive and negative loading directions, respectively, corresponding to a directional difference of 4.2%. Specimen W2off reached peak lateral resistances of 611.8 kN and 621.3 kN, respectively, with a directional difference of 1.5%. These small differences may be associated with material variability and unavoidable asymmetries in specimen loading and crack development. The peak resistances of both specimens were higher than their corresponding design wall shear forces.

**Figure 19:**
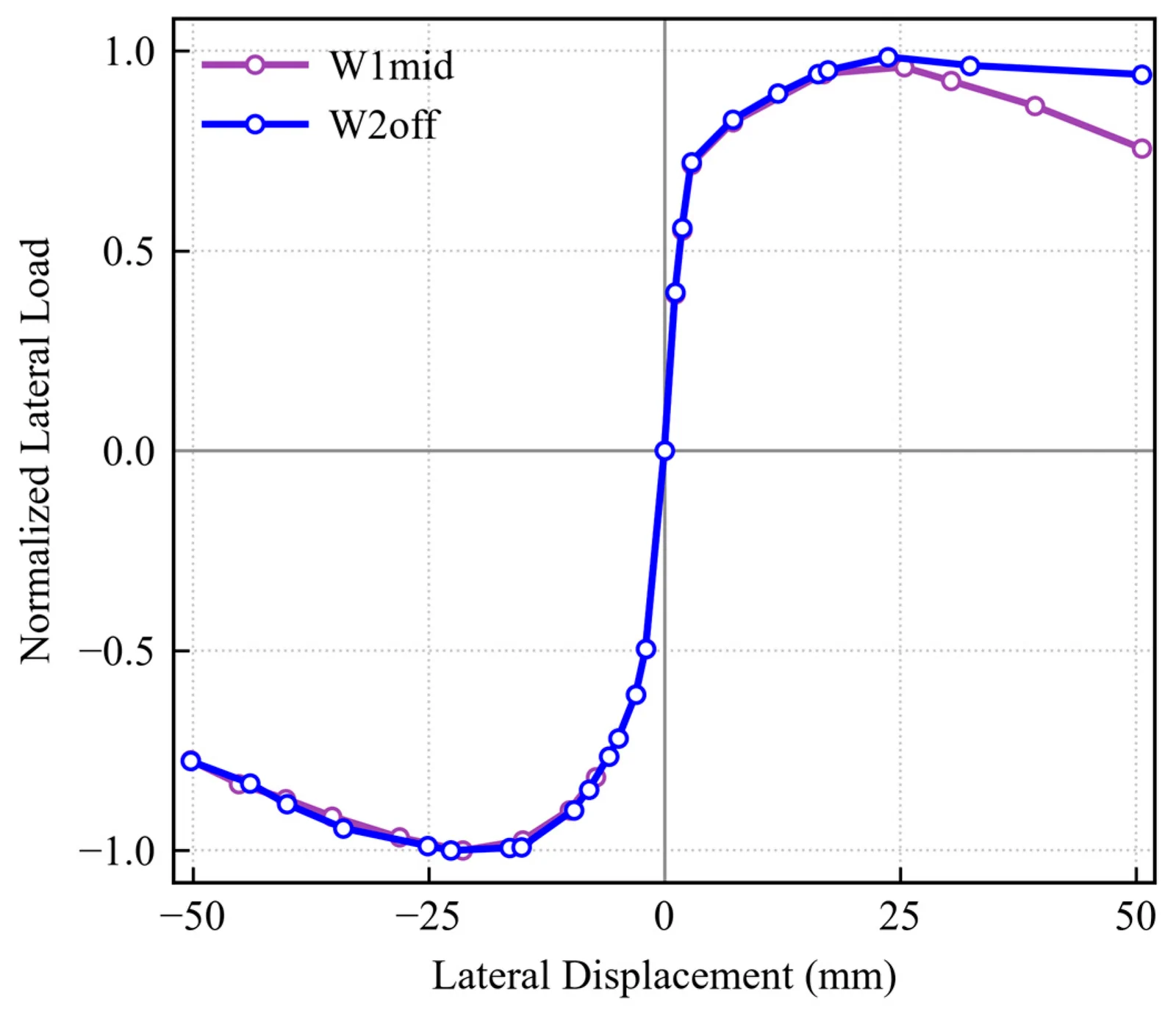
Normalized skeleton curves of wall specimens.

Figure 19 compares the normalized skeleton curves of W1mid and W2off. Since the two specimens had different wall lengths and shear span ratios, the lateral resistance was normalized by the maximum absolute resistance of each specimen to facilitate comparison of the skeleton-curve shape. The normalized skeleton curves show that both specimens had similar ascending branches before reaching the peak resistance. After the peak, W2off exhibited slower strength degradation than W1mid.

**Figure 20:**
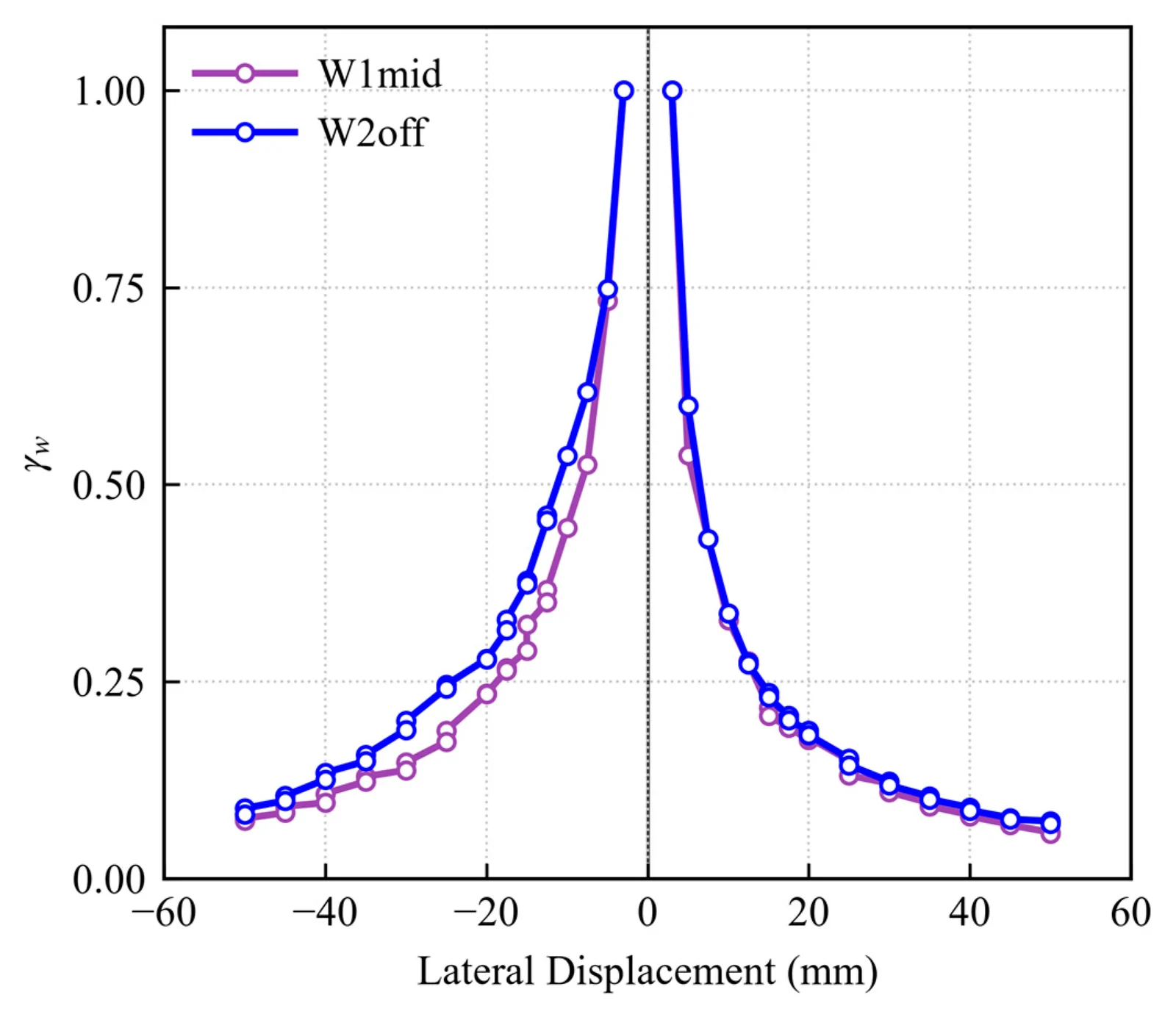
Stiffness degradation of wall specimens.

The stiffness degradation of the specimens is shown in Figure 20. The secant stiffness was calculated separately for the two directions. At the *i*-th loading level, the positive and negative secant stiffnesses were calculated as:

*K_w_*_,*i*_^+^ = |*V_w_*_,*i*_^+^|/|*Δ_w_*_,*i*_^+^|, *K_w_*_,*i*_^−^ = |*V_w_*_,*i*_^−^|/|*Δ_w_*_,*i*_^−^| (11)

where *V_w_*_,*i*_^+^ and *V_w_*_,*i*_^−^ are the positive and negative peak lateral forces at the *i*-th loading level, respectively, and *Δ_w_*_,*i*_^+^ and *Δ_w_*_,*i*_^−^ are the corresponding lateral displacements. The stiffness degradation coefficients in the two loading directions were then defined as:

*γ_w_*_,*i*_^+^ = *K_w_*_,*i*_^+^/*K_w_*_,0_^+^, *γ_w_*_,*i*_^−^ = *K_w_*_,*i*_^−^/*K_w_*_,0_^−^ (12)

where *K_w_*_,0_^+^ and *K_w_*_,0_^−^ are the initial secant stiffnesses in the positive and negative loading directions, respectively.

As shown in Figure 20, both specimens exhibited rapid stiffness degradation at small lateral displacement levels, followed by a slower degradation trend after yielding. Compared with W1mid, W2off generally retained higher stiffness degradation coefficients in both loading directions. This indicates that W2off experienced slower stiffness degradation, which is consistent with its more stable post-peak skeleton response.

## 5. Conclusions

This study experimentally examined the mechanical behavior and effectiveness of a steel-anchor-ring grouted vertical joint, which is a hybrid steel–grout connection intended for precast concrete shear walls. In this connection, steel anchor rings, an inserted vertical bar, and high-strength grout form the primary shear-transfer path between adjacent precast wall panels. The experimental program was carried out at both joint and wall levels, including monotonic direct shear tests, cyclic shear tests under normal tension and compression, and cyclic loading tests on precast shear wall specimens. The main conclusions are summarized as follows:

1. In the direct shear tests, the load–slip behavior, observed interface debonding, and final shank fracture suggested that the grout–panel interface bond contributed substantially to the pre-peak response, whereas anchor-ring mechanical action became increasingly important as bond degradation progressed. In the tested comparisons, the larger anchor-ring size was associated with higher peak and residual resistance, whereas the lower panel concrete strength was associated mainly with reduced interface-bond-related peak resistance.
2. The cyclic shear specimens exhibited distinct responses under the imposed normal-force conditions. The tension–shear specimen showed more pronounced interface opening and bond degradation, together with lower peak resistance and a more slip-dominated response, whereas the compression–shear specimen maintained greater interface contact and exhibited higher peak resistance and more stable post-peak behavior.
3. For the specimens tested, the code-based expression underpredicted the measured peak shear resistance. The measured-to-calculated resistance ratios were 1.97–2.70 for the direct shear and compression–shear specimens, but decreased to 1.20–1.31 for the tension–shear specimens due to interface opening and bond degradation.
4. The wall-level demand analysis showed that the shear demand transferred across the vertical joint was affected by both the joint location and the wall shear span ratio. A larger shear span ratio led to a higher calculated joint shear demand. In addition, moving the vertical joint from the wall mid-length to an offset position reduced the joint shear demand by 25.8%. These results indicate that the vertical-joint layout and wall geometry should be considered in design, rather than treating the joint only as a construction arrangement.
5. The wall-level cyclic tests showed that, within the tested configurations, the vertical joints maintained shear transfer without severe separation or connector fracture and did not govern the global failure. Both specimens ultimately failed through flexural damage at the wall base. The specimen with a calculated joint demand below the code-based resistance exhibited limited joint-related cracking, stable hysteretic behavior, slower post-peak strength degradation, and better stiffness retention.

## 6. Limitations and Future Research

Although the present study provides experimental evidence for the proposed steel-anchor-ring grouted vertical joint, several limitations should be recognized. First, the joint-level experiments included five specimens, with each configuration represented by a single specimen. The limited parameter range and lack of replicate specimens prevented characterization of experimental variability or establishment of a statistically reliable lower-bound resistance. Moreover, the cyclic tests employed a loading protocol with only a limited number of repeated cycles. The reported cyclic results should therefore be regarded as a preliminary characterization under the tested loading history rather than as seismic qualification of the connection. In addition, local strains and connector-force redistribution in the anchor-ring connections were not measured. Consequently, the respective contributions of grout–panel interface bond and anchor-ring mechanical action could not be quantified directly, and the proposed force-transfer mechanism was inferred from the load–slip behavior and observed damage sequence. Future investigations will expand the experimental database by incorporating replicate specimens and broader variations in joint configurations and normal-force levels. More extensive repeated-cycle and variable-amplitude loading protocols, together with variable normal-force histories, will be adopted to evaluate cumulative damage, cyclic stability, and deformation capacity more comprehensively. Local strain and full-field deformation measurements will be used to quantify the respective contributions of the grout–panel interface bond and anchor-ring mechanical action. Based on the expanded experimental database, numerical models will be developed and assessed against the test results to identify the governing parameters, examine the applicability of existing design equations, and support the development of mechanics-based shear-resistance models.

Second, the wall-level test program did not include monolithic or conventional-joint control walls, and both tested specimens developed wall-base flexural failure before a joint-controlled response occurred. Consequently, the present tests only demonstrate that the proposed vertical joints remained functional, maintained shear transfer, and did not govern failure under the tested demands. They do not establish equivalent or improved capacity, stiffness, ductility, or energy dissipation relative to monolithic or conventional-joint walls, nor do they define the ultimate resistance and deformation limits associated with joint failure. Future studies will include appropriate monolithic and conventional-joint control walls, together with specimens proportioned to develop joint-controlled response while avoiding premature wall-base failure, enabling direct performance comparisons and clearer assessment of the joint contribution to the global wall response.

Third, the specimens were fabricated and assembled under controlled laboratory conditions, whereas field implementation may involve variations in interface roughness, moisture conditions, surface contamination, workmanship, grout-filling quality, and installation accuracy. Future full-scale assembly and tolerance studies will quantify permissible sleeve-position and panel-erection deviations, minimum grout-access and operation-opening requirements, installation time, inspection procedures, and construction cost, thereby providing a basis for allowable tolerances and quality-control requirements.

Finally, the in-service performance of the connection can be affected by environmental exposure, repeated service loading, and elevated temperatures [52]. Future studies will investigate the durability, fatigue, and fire performance of the connection and provide a basis for appropriate protection, inspection, and maintenance measures.

## Figures and Tables

**Table 1:** Comparison of vertical joint constructability.

Joint Type	Wet Joints	Dry Joints	Existing Hybrid Joints	Proposed Joint
Ref.	[29,30,31,32,33,34,35,36,37,38,39]	[40]	[41]	-
No side-face projecting bars	×	✓	✓	✓
Large wet region avoided	×	✓	✓	✓
Distributed shear-transfer path	✓	×	×	✓
Post-erection connector adjustment	×	×	×	✓

Note: ✓ indicates that the corresponding criterion is satisfied; × indicates that it is not satisfied.

**Table 2:** Design details of joint-level specimens.

Specimen	Test Type	Normal-Force Condition	Normal Force *N_j_* (kN)	Anchor-Ring Arrangement	Panel Concrete Grade
DS16-C40	Direct shear	None	0	M16@500	C40
DS20-C40	Direct shear	None	0	M20@500	C40
DS16-C30	Direct shear	None	0	M16@500	C30
TS16	Cyclic shear	Tension	−20	M16@500	C40
CS16	Cyclic shear	Compression	40	M16@500	C40

**Table 3:** Measured mechanical properties of steel anchor rings and inserted bars.

Material	Size	Steel Grade	Yield Strain	Yield Strength (MPa)	Ultimate Strength (MPa)
Steel Anchor Ring	M16/M20	45#	0.0023	455.2	655.4
Reinforcement	D14	HRB400	0.0024	477.5	642.5

**Table 4:** Comparison between measured and calculated shear resistance.

Specimen	Normal-Force Condition	Normal Force *N_j_* (kN)	*V_j_*_,c_ (kN)	*V*_max_ (kN)	*η_j_*
DS16-C40	Pure shear	0	110	297	2.70
DS20-C40	Pure shear	0	172	339	1.97
DS16-C30	Pure shear	0	110	257	2.34
TS16^+^	Tension–shear	−20	94	123	1.31
TS16^−^	Tension–shear	−20	94	113	1.20
CS16^+^	Compression–shear	40	142	355	2.50
CS16^−^	Compression–shear	40	142	330	2.32

**Table 5:** Shear checks of vertical joints in wall specimens.

Specimen	*L_w_* (mm)	*d_j_* (mm)	*L_j_* (mm)	Anchor-Ring Arrangement	*V_j,d_* (kN)	*N_j_* (kN)	*V_j,c_* (kN)	*η_j_V_j,c_* (kN)	*V_j,d_* ≤ *V_j,c_*	*V_j,d_* ≤ *η_j_V_j,c_*
W1mid	1000	500	2800	M20@250	1053	4.4	862	1698	Fail	Pass
W2off	2000	450	2800	M20@250	784	−17.3	844	1663	Pass	Pass

**Table 6:** Mechanical properties of steel anchor rings and reinforcement in wall cyclic tests.

Material	Size	Steel Grade	Yield Strain	Yield Strength (MPa)	Ultimate Strength (MPa)
Steel Anchor Ring	M20	45#	0.0023	455.2	655.4
Reinforcement	D8	HRB400	0.0024	485.0	682.5
Reinforcement	D12	HRB400	0.0025	492.5	650.0

## Data Availability

The original contributions presented in this study are included in the article. Further inquiries can be directed to the corresponding author.
